# Supplementary material for: Micro𝕊plit: semantic unmixing of fluorescent microscopy data
Source: Nat Methods. 2026 May 5;23(5):1047–57. doi: 10.1038/s41592-026-03082-1 (PMC13167470; doi:10.1038/s41592-026-03082-1)
Supplement: Supplementary file 1 — Supplementary Notes 1–4, Tables 1–12 and Figs. 1–54. [file 41592_2026_3082_MOESM1_ESM.pdf]

nature methods

Supplementary information https://doi.org/10.1038/s41592-026-03082-1

Micro𝕊plit: semantic unmixing of fluorescent microscopy data

In the format provided by the authors and unedited

Supplementary Material

MicroSplit: Semantic Unmixing of Fluorescent Microscopy Data

Ashesh Ashesh[1], Federico Carrara[1,2], Igor Zubarev[1], Vera Galinova[1], Melisande Croft[1], Melissa Pezzotti[3], Daozheng Gong[4], Francesca Casagrande[1], Elisa Colombo[1], Stefania Giussani[1], Elena Restelli[1], Eugenia Cammarota[1], Juan Manuel Battagliotti[1], Nikolai Klena[1], Moises Di Sante[3], Raghabendra Adhikari[5], Daniel Feliciano[5], Gaia Pigino[1], Elena Taverna[1], Oliver Harschnitz[1], Nicola Maghelli[1], Norbert Scherer[4], Damian Edward Dalle Nogare[1], Joran Deschamps[1], Francesco Pasqualini[3], Florian Jug[1*]

[1]Fondazione Human Technopole, V.le Rita Levi-Montalcini, 20157, Milan, Italy.
[2]Università Campus Bio-Medico di Roma, Via Álvaro del Portillo 21, 00128, Rome, Italy.
[3]University of Pavia, Corso Strada Nuova, 65, 27100, Pavia, Italy.
[4]University of Chicago, 5801 S Ellis Ave, 60637, Chicago, USA.
[5]HHMI/Janelia Research Campus, 19700 Helix Drive, Ashburn, 20147, VA, USA.

*Corresponding author(s). E-mail(s): florian.jug@fht.org;
Contributing authors: ashesh.ashesh@fht.org;

1 Analyzing Factors that Affect Predictive Performance

1.1 Pixel-noise (pixel-wise independent noise)

From our different experiments, we have found that there are two prominent factors that affect the performance on semantic unmixing tasks. The first factor is the amount of pixel-independent noise that is present in the superimposed input images and target images. More noise means inferior performance. To quantify the effect of noise, we imaged our HT-LIF24 dataset with exposure durations of $2ms$, $3ms$, $5ms$, $20ms$, and $500ms$. We imaged it such that the underlying content in these sub-datasets is identical. That is, for every frame in the $2ms$ acquisition, we have the corresponding higher SNR frames in $3ms$, $5ms$, $20ms$ and $500ms$ acquisitions. We trained a three-channel semantic unmixing task separately for each exposure duration sub-dataset. We made predictions on the held-out test set input frames from their respective exposure duration sub-datasets. We evaluate the prediction against the target channels present in the $500ms$ sub-dataset.

Although the results (Tasks XXIV-XXIX) in Table 1 show the expected trend (semantic unmixing quality decreases when using lower SNR training data), even the shortest exposure time of $2ms$ still lead to unmixed predictions that are fit for downstream processing and analysis (in all cases we measured a PSNR > 30.8 and $\mathbb{M}$icroMS-SSIM > 0.94). To explain the performance drop, Extended Data Figure 2 shows that reduced PSNR primarily results from the loss of high-frequency details.

1.1.1 Lessons learned from the difficult Task XXIII (on HHMI-D25 data)

Out of all tasks mentioned in the Table 1, the task XXIII which uses HHMI-D25$_{8bit}$ dataset has considerable performance issues, especially in the third channel (see Supplementary Figure 52). In the next few paragraphs we will describe our approach to investigating this issue and present a working solution. We do this to provide an example for how users of Micro$\mathbb{S}$plit can improve solutions that might initially not lead up to the required semantic unmixing quality.

Signal-to-noise (SNR) is one of the factors which plays a role in almost all deep-learning based methods and denoising approaches, and semantic unmixing is no exception. To investigate the role of SNR for task XXIII, we first denoised the raw data of HHMI-D25$_{8bit}$ using Noise2Void [4], and then trained Micro$\mathbb{S}$plit using those denoised images, calling this training task 'Task XXXI'. Comparing the results of tasks XXIII and XXXI, see also Supplementary Table 1, it becomes apparent that prior denoising has a rather strong positive effect on the quality of the achieved semantic unmixing results ($>$ 7db PSNR improvement), suggesting that the low SNR in the original data might indeed have caused the bad performance.

Since HHMI-D25$_{8bit}$ is stored in unsigned int8, meaning that pixel values are in $[0, 255]$, there are actually only a few distinct integer values presenting most of the data. Denoising this data, besides increasing the SNR, also increases the number of unique pixel values. We hypothesized that in addition to SNR, an overly discreet nature of pixel values might also be detrimental to semantic unmixing. To test this hypothesis, we imaged the HHMI-D25$_{16bit}$ data subset, where the unsigned int16 format was used to store the data, increasing the pixel intensity range to $[0, 65535]$. Doing so, we ensured that the SNR (ratio of average foreground value to average background value) is as similar as possible for both, the 8 and 16 bit versions of the HHMI-D25 data. Using the 16bit data did indeed improve the quality of predictions, also for the problematic third channel, as can be seen in Supplementary Figure 53 and Extended Data Figure 3. The quantitative metrics in Table 2, however, do not capture the improvement we can perceive by comparing those figures. To fully validate our hypothesis regarding SNR and overly discrete pixel intensities, we imaged another data subset of HHMI-D25, namely HHMI-D25$_{16bit,0.25}$, which not only uses unsigned uint16 format but also bins four pixels into one (thereby increasing SNR on the cost of spatial resolution). On this data subset we defined Task XXXVI, and indeed observe a much improved semantic unmixing performance (see Table 2 and Figure 54).

We also experimented with synthetic Gaussian and Poisson noise with HHMI-D25 dataset versions. The motivation was to start from a working setup, make the training and evaluation dataset noisy and inspect the performance degradation. For this purpose we picked HHMI-D25$_{16bit}$ and HHMI-D25$_{8bit,denoised}$. We added Gaussian noise (σ) and Poisson noise (λ). Given an image x, its noisy version can be expressed $Poi(x/\lambda) \cdot \lambda + \epsilon$, where $\epsilon \sim \mathbb{N}(0, \sigma)$ and $Poi()$ represents the Poisson distribution with parameter λ. As it was to be expected, the performance degrades with noise (see Tasks XXXII, XXXIV, and XXXV; Extended Data Figures 3, and 4, and a quantitative comparison in Supplementary Table 2).

1.1.2 Out-of-distribution SNR

We also use the set of models trained on different HT-LIF24 sub-datasets to understand how the performance degrades with out-of-distribution inputs. For this, we evaluate the performance of Micro𝕊plit trained on one exposure duration on the superimposed input images coming from a different exposure duration. We present the results in Figure 1. The different curves represent individual Micro𝕊plit models trained on one specific exposure duration sub-dataset as specified in the legend. On the x-axis, we have different evaluation sub-datasets, referred to by their exposure duration. From CARE-PSNR and 𝕄icroMS-SSIM plots, one can observe that performance improves as one increases the exposure duration. Additionally, upon observing performance on $2ms$ and $500ms$ sub-datasets, one can see that in most cases, the larger the difference between the exposure duration of the training sub-dataset and evaluation sub-dataset, the higher the performance drop. For instance, if we look at CARE-PSNR plot, the two worst-performing Micro𝕊plit models on $2ms$ acquisition were trained on $20ms$ and $500ms$. And the two worst-performing Micro𝕊plit models on $500ms$ acquisition were trained on $2ms$ and $3ms$. On a different note, one can observe much less variation in SSIM and MS-SSIM plots. We discuss this aspect in Section 3.

1.2 Spatial Correlation

The structures present in the cell are spatially correlated. For example, nuclei are typically in the central regions of cells, while the cell boundary is, by definition, on the boundary of a cell. The knowledge about the cell surface, therefore, can tell something about where the nucleus should or should not be present. We wanted to understand how important this spatial correlation is for our semantic unmixing task.

For this, we worked with the Pavia-P23 dataset where we modified the input patch process during training. The default method is to pick a random location in a frame, extract patches for both structures (target channels) from that location, and sum them to create superimposed input (*i.e. Training Mode I*). Using *Training Mode I*, the spatial correlation between the imaged structures is naturally maintained. To disrupt this, we conducted experiments with the following alterations.

In the first alteration, we kept *Training Mode I* for 50% of all training patches, but created the other 50% of training patches by picking two different random locations and adding them together to create an input patch (*i.e. Training Mode II*). Hence, we maintain sound spatial correlations between the structures to be unmixed in half the training data. In the second alteration, we create all training data patches according to *Training Mode II*, thus eliminating all spatial correlations between the structures that should be unmixed and forcing the trained network to rely fully on the structural appearance of the structures only.

We report the metrics CARE-PSNR and 𝕄icroMS-SSIM in Table 9 which shows that the absence of spatial correlation indeed results in a drop in performance by $0.3 - 0.5$ dB PSNR.

1.3 Similarity of Structures to be Unmixed

Since our method relies heavily on the spatial appearance of the structures to be unmixed, we wondered how dissimilar two structures have to be to lead to good semantic unmixing results. Hence, it is best to image structures in single channels that are as dissimilar as possible.

In μSplit [6], we proposed a synthetic semantic unmixing dataset based on combinations of sinusoidal curves that required longer-range structure integration. Although the structures were very simple, the network without using LC was unable to split the input and ended up only predicting the input for both target channels equally.

However, to better evaluate to what extent a network can separate structures that have a similar appearance, we designed the following experiments. Using the microtubule channel of the HT-LIF24 dataset, we created a 2-channel splitting task by mixing patches from the microtubule channel with other patches from the exact same channel. We reasoned that the network will not be able to split structures that are literally taken from the same set of data (see bottom row in Extended Data Figure 5). To give the network a chance, we then started to alter one of the two superimposed copies by scaling the data (uppermost four rows in Extended Data Figure 5). This leads to the superposition of data that is structurally still very similar, but one copy will have a slightly larger appearance.

In Extended Data Figures 5 and 6 we show the results of these experiments, conducted with a range of different relative scaling between the two copies of the microtubule data. The results clearly show that the network is capable of semantic unmixing the structures in all cases, even if the scaling factor was as low as 1.125. Note also that it takes the network increasingly longer before the splitting performance starts lifting

off and reaching its peak, as can be inferred from the inflection point and convergence behavior seen in the PSNR *vs.* training steps plot in Extended Data Figure 6.

1.4 Unequal Channel Intensities

Another factor that plays a key role in the performance of Micro𝕊plit is the skewness in intensity between different target channels in the superimposed input image. This effect can be caused by diverging fluorophore densities for different structures, by fluorophore bleaching, or by using inadequate laser lines and/or laser power settings for some fluorophores. Any of this can lead to acquisitions where one or more of the structures are only weakly present in the superimposed input. We hypothesized that predictions for the brighter (dominant) structure should still be of good quality but that the quality for the dim structure might be worse.

To test this hypothesis, we worked with the 2-channel semantic unmixing tasks from the Pavia-P24 dataset, where we varied the laser power for the two channels we imaged from a balanced setting (50:50) to increasingly more skewed settings (*i.e.* 66:33 and 84:16). Note that the total laser power was kept the same. We acquired images at these three levels of skewness at three different total laser powers and resulting signal-to-noise ratios, giving us a total of 9 sets of image acquisitions. We show the results of all semantic unmixing experiments in Table 3, where 'Skew' denotes the asymmetry in the power distribution ('Balanced', 50:50; 'Mid', 66:33; 'High', 84:16).

We found that, across three total laser power levels, the performance of the bright first channel generally increases as we go from Balanced to High skew. The second dim channel shows the inverse behavior. Note that since the samples we imaged at different imaging settings were not the same, each experiment is based on a different body of training and evaluation data. This causes additional fluctuations in the performance metrics we report and makes the presented results to be non-monotonic. If all 9 imaging sessions had captured the same samples, we would expect the results to show a monotonic trend.

Unequal Channel Intensities - Two Extreme Examples

We work with two more examples to investigate this case. For the first example, we worked with the nucleus and tubulin channels of the Chicago-Sch23 dataset. As always, we create the superimposed input by summing the two channels. Here, the nucleus is very weak (the channels being very skewed in their relative intensity), so much so that nuclei are de facto invisible to the naked eye. However, Micro𝕊plit was still able to unmix these channels at a reasonable quality. One can observe the superimposed input, the two targets, and the predictions of Micro𝕊plit in Supplementary Figure 3.

For a second example, we inspect Task II which uses the HT-P23A dataset. Here, the two structures are microtubules and nuclei, with the latter being the weaker channel. Similarly to the first example, the weaker nucleus channel is effectively invisible in superimposed images, as can be seen in Supplementary Figure 4. However, unlike in the previous example, the structure of nuclei in this dataset is less regular and more variable. Due to this and in light of the high uncertainty caused by the highly skewed channel intensities, Micro𝕊plit MMSE predictions for the nucleus channel become rather blurry, as can be seen in Supplementary Figure 4. Still, in many practical applications, *i.e.* every time the detailed texture of the nuclei is not important, such predictions can still be sufficient for the desired data analysis to be carried out.

1.5 Sufficient Lateral Image Context

Our method combines the benefits of μSplit and denoi𝕊plit. One of the benefits of μSplit architecture we demonstrated in [6] is its ability to utilize additional surrounding spatial context for a given superimposed input patch through LC inputs and its ability to employ much deeper architectures. In [6], we showed that having sufficient spatial context helps with relatively large structures spanning hundreds of pixels.

2 Details on Uncertainty Quantification and Calibration

In this section, we detail our uncertainty estimation and calibration module. Our approach builds on the methodology of denoi𝕊plit [7], originally inspired by [15], with a modification to the calibration process as described below. Our variational approach is inherently capable of sampling, meaning it can generate slightly different outputs for the same input each time a prediction is made. We leverage this property to produce multiple predictions for a single input, resulting in several predicted values per pixel. Using these, we calculate the standard deviation for each pixel. To ensure these pixel-wise standard deviations

Training Mode	Correlation between structures preserved?	Input data is…	Note
Training Mode I	Yes	…mixed computationally.	Existing imaging data can be used (no special acquisitions required).
Training Mode II	No	…mixed computationally.	Existing imaging data can be used (no special acquisitions required). Results might be of lesser quality if reliable structural correlations between channels to be unmixed do exist.
Training Mode III	Yes	…imaged directly.	Mixed input must directly be imaged along with the individual target channels. Can lead to best performance when imaging is done well, but requires additional microscopy work.

Table 1 Overview of Training Modes: Properties and key advantages/ disadvantages of the training modes we propose. We say that the correlation between structures is preserved when their relative positioning in the computationally mixed input maintains their biologically occurring relative positioning in the sample.

Task	Dataset	Synthetic Noise	PSNR			MicroMS-SSIM		
XXIII	HHMI-D25$_{8bit}$	-	22.5	31.3	24.3	0.840	0.768	0.793
XXXI	HHMI-D25$_{8bit,denoised}$	-	37.9	35.2	38.6	0.990	0.903	0.974
XXXII	HHMI-D25$_{8bit,denoised}$	$\sigma = 20, \lambda = 30$	31.8	27.3	32.4	0.939	0.664	0.859
XXXIII	HHMI-D25$_{16bit}$	-	23.2	27.9	24.8	0.772	0.849	0.779
XXXIV	HHMI-D25$_{16bit}$	$\sigma = 2K, \lambda = 5K$	23.2	27.9	24.8	0.827	0.861	0.778
XXXV	HHMI-D25$_{16bit}$	$\sigma = 4K, \lambda = 10K$	23.0	27.4	24.6	0.746	0.838	0.700
XXXVI	HHMI-D25$_{16bit,0.25}$	-	32.4	32.2	35.0	0.990	0.929	0.991

Table 2 Performance of MicroSplit on sub-datasets of HHMI-25. This table presents quantitative results of MicroSplit across various tasks defined on parts of the HHMI-25 dataset. While the original Task XXIII on HHMI-D25$_{8bit}$ does not lead to satisfying predictions, mainly for channel 3 (see Supplementary Figure 52), tasks on similar data with higher SNR (Task XXXI, see Extended Data Figure 4, row 3), increased pixel diversity (Task XXXIII, see Extended Data Figure 3, row 2), or both (Task XXXVI, see Supplementary Figure 54) demonstrate notably improved semantic unmixing performance. Tasks XXXII, XXXIV, and XXXV are identical to Tasks XXXI and XXXIII, respectively, but with added synthetic noise, simply to demonstrate how the lower SNR drops the unmixing performance achievable with MicroSplit.

Skew	Channel 1 Laser Power			Channel 2 Laser Power		
	High	Mid	Low	High	Mid	Low
Balanced	24.3	23.1	21.9	29.9	24.3	23.0
Mid	28.2	24.0	22.9	25.6	22.3	21.7
High	25.2	24.3	23.3	24.1	22.4	22.8

Table 3 Varying the laser power and skew with Pavia-P24 dataset. Skew column denotes the relative importance given to channel 1. High skew means larger laser power allocated to Channel 1 compared to Channel 2

Model	PSNR	SSIM
Training Mode I: input = $C_1 + C_2$	35.9	0.956
Training Mode II: input = $C_1 + C_2$ (shuffled)	34.6	0.947
Training Mode III: input comes from microscope	36.5	0.960

Table 4 Performance comparison for different Acquisition Modes. We use the Sox2 vs Golgi splitting task of the HT-T24 dataset for this purpose. For Acquisition Mode I and II, real input image is not used during training. Instead, input is created by synthetically summing the two target channels. In all cases, evaluation is done on the held-out test set using the real input channel, that is, on the input which is not synthetic and comes directly from the microscope.

	C1				C2			
	(2D) Z=1	Z=5	Z=9	Z=15	(2D) Z=1	Z=5	Z=9	Z=15
CARE-PSNR	35.8	38.7	39.5	39.7	30.9	33.7	34.5	34.7
MicroSSIM	0.865	0.878	0.885	0.886	0.729	0.757	0.772	0.767
MicroS3IM	0.950	0.970	0.973	0.974	0.929	0.950	0.956	0.956

Table 5 Performance improvement with 3D models on HT-H24 dataset. As we increase the number of z-slices fed to the model, we see the performance improve in all our metrices.

	Set I			Set II		
	C_1	C_2	C_3	C_1	C_2	C_3
Input C_1	0.50	0.33	0.17	0.625	0.25	0.125
Input C_2	0.33	0.50	0.33	0.25	0.625	0.25
Input C_3	0.17	0.33	0.50	0.125	0.25	0.625

Table 6 We use the weights to mix the three channels C_1, C_2 and C_3.

Model	Channel 1			Channel 2			Channel 3		
	A	B	C	A	B	C	A	B	C
PICASSO Set I	24.1	0.633	0.751	24.1	0.898	0.863	31.6	0.858	0.914
PICASSO Set II	24.2	0.638	0.757	24.1	0.904	0.864	32.2	0.841	0.911
Micro𝕊plit	**33.9**	**0.730**	**0.902**	**34.9**	**0.909**	**0.884**	**38.21**	**0.910**	**0.958**

Table 7 Quantiative comparison with PICASSO. A: RI-PSNR, B: MicroSSIM, C: MicroS3IM

correspond closely to the actual prediction errors, we apply a straightforward linear scaling. Opting for a linear transformation, rather than a more complex method, helps minimize the risk of overfitting. This simple linear transformation based approach allows us to easily estimate pixel-wise errors for a test input: we simply run multiple predictions, compute the standard deviation for each pixel, and then apply the learned linear transformation. Importantly, this process only requires the test input, the trained model, and the parameters of the linear transformation.

Next, we provide a technically accurate description of the methodology. For each input image x (of dimensions $H \times W$), we generate $k = 50$ predictions. At every pixel location p in channel i, this gives us k predicted values, which we use to compute the pixel-wise standard deviation $\sigma_i[p]$. Next, for calibration, for each channel i, we sort the computed pixel-wise standard deviations and bin them over $l = 30$ equally sized bins. Specifically, we implement the above-mentioned logic by storing pixel location p of the relevant pixels in these bins $\{B_i^1, B_i^2 \dots B_i^l\}$. We then compute the root mean variance (RMV) and root mean squared error (RMSE) for each channel i and bin j as

$$\mathrm{RMV}_i(j) = \sqrt{\frac{1}{|B_i^j|}\sum_{p \in B_i^j} \sigma_i[p]^2} \quad \text{, and} \tag{2}$$

$$\mathrm{RMSE}_i(j) = \sqrt{\frac{1}{|B_i^j|}\sum_{p \in B_i^j} (c_i[p] - \hat{c}_i[p])^2}, \tag{3}$$

where c_i and $\hat{c}_i$ represent the ground truth image and the estimated image for the ith channel respectively. Subsequently, we fit a linear relationship between RMSE and RMV for each channel i, learning the coefficients α_i and β_i that minimize the objective

$$\arg\min_{\alpha_i, \beta_i} ||\mathrm{RMSE}_i - (\alpha_i \cdot \mathrm{RMV}_i + \beta_i)||_2^2. \tag{4}$$

Note, that denoi𝕊plit employed pixel-level regression instead of bin-based regression. However, that method has a key limitation: since images often contain many background pixels, the regression tends to be biased toward these regions, resulting in poor calibration for foreground pixels, which is problematic.

During evaluation, we sample multiple predictions for a given input. For a channel i, we compute the pixel-wise standard deviation σ_i over the multiple samples. The estimate of pixel-wise RMSE is given by $\mathrm{RMSE}_i = \sigma_i * \alpha_i + \beta_i$. It is worth noting that the estimation of α_i and β_i is done using the validation data and evaluation for the calibration is done on the test data.

Model	Channel 1			Channel 2			Channel 3		
	A	B	C	A	B	C	A	B	C
PICASSO Set I	.779	.021	.020	.587	.007	.012	.454	.026	.005
PICASSO Set II	.779	.032	.019	.587	.007	.012	.478	.038	.006
MicroSplit	.839	.025	.008	.475	.005	.010	.613	.012	.003

Table 8 Standard error table for Table 7. A: CARE-PSNR, B: MicroSSIM, C: MicroMS-SSIM

Corr.	Balanced				Mid-Skew				High-Skew			
	PSNR		MicroMS-SSIM		PSNR		MicroMS-SSIM		PSNR		MicroMS-SSIM	
0%	23.9	29.5	0.594	0.731	27.7	25.2	0.755	0.656	24.9	23.8	0.695	0.552
50%	24.1	29.6	0.614	0.761	28.1	25.5	0.761	0.657	25.1	23.9	0.781	0.699
100%	24.3	29.9	0.682	0.839	28.2	25.6	0.780	0.696	25.2	24.1	0.722	0.623

Table 9 Here, we inspect the importance of spatial correlation in the structures. In row 1, input is created by summing patches taken from different locations. In row 2, for 50% of the time, input is created by summing patches taken from different locations. In the remaining 50% of times, input is created by summing patches taken from the same location. In row 3, all training input is created by always summing together co-located patches. Evaluation is done on co-located patches.

	Laser Power % (50/50)		Laser Power % (33/66)		Laser Power % (16/84)	
	446nm	477nm	446nm	477nm	446nm	477nm
SNR: high	40	40	27	53	13	67
SNR: mid	20	20	13	27	7	33
SNR: low	10	10	7	13	3	17

Table 10 Laser power distribution in the several sub-datasets of Pavia-P24 dataset

With MicroSplit, we have presented a simple, yet effective way to estimate pixel-wise uncertainty estimates and evaluate the calibration of the predicted uncertainty. One of the advantages of the presented calibration procedure is that it does not alter the original predictions but instead learns a mapping that best predicts the measured error.

3 Evaluation Metrics

SSIM (Structural Similarity Index Measure) [25, 26] and PSNR (Peak Signal-to-Noise Ratio) are one of the most popular metrics used in regression tasks. While MSE-based metrics like PSNR estimate pixel-wise distance between the target and the prediction, SSIM captures structural similarity. For large images that may have sub-regions having different structural characteristics, people found it useful to work with MS-SSIM (Multi-Scale SSIM) [27] instead of SSIM. Since microscopy images are typically large in pixel dimensions, MS-SSIM therefore seemed a better alternative over SSIM. However, our earlier work showed that when working with fluorescence microscopy data, especially with differing exposure durations, PSNR, SSIM, and MS-SSIM are all ill-suited [8]. Among several issues presented in the above-mentioned work, here we illustrate the issue most relevant to MicroSplit. The issue is that the input to MicroSplit is typically a noisy image which means that it was imaged using a small exposure duration. This means that the image covers a smaller portion of the full dynamic range. In other words, the pixel intensities found in the input image are relatively low in magnitude. Since the network was trained with noisy target images, which had a similar dynamic range, the prediction by the network also covers a similar amount of dynamic range. However, for the HT-LIF24 dataset, we have the corresponding high SNR target, which covers a much larger portion of the dynamic range and so has larger pixel values. So, the problem is: how to compare a prediction having a low-dynamic range with a high-dynamic range image? The core idea in the solution used in CARE-PSNR [3] and MicroMS-SSIM [8] is to estimate an optimal linear transformation for the prediction. This linear transformation makes the pixel intensity values of the scaled prediction as close to the corresponding target channel as possible. The relevant metric (PSNR, SSIM, or MS-SSIM) is then computed between the target and the scaled prediction. We have used CARE-PSNR and MicroMS-SSIM in this work.

Multiple Z-stacks of 6 planes with a step size of 1 µm were acquired to populate the dataset for each condition.

4 Further Details on all Experiments (Learning Tasks)

In this section, we mention specific details about individual tasks mentioned in the Table 1. Unless explicitly specified, we use the same hyperparameters for all tasks. Please refer to the code for details on the hyperparameters.

4.1 Two-channel semantic unmixing tasks

Task I

It is created from the HT-H24 dataset. It works on 3D z-stacks. The acquisition mode used in this dataset was *Training Mode I*. We show one qualitative example in Supplementary Figure 13.

Task II

It is created from HT-P23A dataset. It works on 3D z-stacks. The acquisition mode used in this dataset was *Training Mode I*. We show one qualitative example in Supplementary Figure 14.

Task III

It is created from the HT-P23B dataset. It works on 3D z-stacks. The acquisition mode used in this dataset was *Training Mode I*. We show one qualitative example in Supplementary Figure 15.

Task IV-XII

These tasks are created from the Pavia-P24 dataset. The acquisition mode used in them was *Training Mode III-a*. These tasks work on 2D frames. There are nine different acquisitions within this dataset that differ in (a) the laser power distribution among the two target channels and (b) the overall SNR. SNR has three levels, namely low, mid, and high, with low having the lowest SNR and high having the highest SNR level. The SNR level fixes the total combined laser power used for both channels. Within a single SNR level, we distribute the power among the two channels in three ways, namely, `50/50`, `66/33`, and `84/16` denoting the percentage laser power allocation to the respective channel. So, `84/16` means 84% of the total laser power was allocated to channel 1 and 16% was allocated to channel 2. We show one qualitative example per task in Supplementary Figures 22, 24, 23, 25, 26, 27, 28, 29, 30.

Task XIII

This task was created from the HT-T24 dataset. It works on 2D frames. The acquisition mode used in them was *Training Mode III-a*. We show one qualitative example in Supplementary Figure 31.

Task XIV

This task was created from the HT-LIF dataset. It used (Nucleus) and Microtubules as the two channels. It works on 2D frames. The acquisition mode used in them was *Training Mode III-a*. This task worked with the sub-dataset that had the exposure duration of $5ms$. We show one qualitative example in Supplementary Figure 32.

Task XV-XX

These tasks were created from the Chicago-Sch23 dataset. The Chicago-Sch23 dataset has four structures, and these tasks capture all possible 2-channel semantic unmixing tasks from these four structures. These are Structured Illumination Microscopy (SIM) images, and due to the computational post-processing that happens in SIM, the resulting images have very different noise characteristics. Therefore, we disabled the use of noise models for all tasks generated from the Chicago-Sch23 dataset. More specifically, in Equation 1, we set $w = 0$. We show one qualitative example per task in Supplementary Figures 16, 17, 18, 19, 20, 21.

4.2 Three-channel semantic unmixing tasks

Task XXI

This task is a 3-channel semantic unmixing task generated from the CBG-Z18 dataset. This is a 3D dataset and so, we employ the 3D version of Micro$\mathbb{S}$plit. We show one qualitative example in Supplementary Figure 11.

Task XXII

This task is a 3-channel semantic unmixing task generated from the CBG-N18 dataset. This is a 3D dataset and so, we employ the 3D version of Micro𝕊plit. We show one qualitative example in Supplementary Figure 12.

Task XXIII

This task is a 3-channel 3D semantic unmixing task generated from HHMI-D25 dataset. Specifically, mitochondria, lysosomes and nuclei channels from HHMI-D25$_{8bit}$ sub-dataset is used to create this task. This is a 3D dataset and so, we employ 3D version of Micro𝕊plit. We show one qualitative example in Supplementary Figure 52.

Tasks XXIV-XXVIII

These tasks are generated from the HT-LIF24 dataset which has four structures. The three structures picked for these tasks are Nucleus, Microtubules and Kinetocore. While these tasks aim at splitting apart the above-mentioned three structures, they differ in the exposure duration of the training and evaluation dataset. The exposure duration used is mentioned in *Task Details* column of Table 1.

We show one qualitative example per task in Supplementary Figures 8, 9, 10, 6, 7.

Task XXXI, XXXII

These two tasks are 3-channel 3D semantic unmixing tasks generated from the denoised version of HHMI-D25$_{8bit}$ dataset, with denoising done by Noise2Void [4]. Mitochondria, lysosomes and nuclei channels from HHMI-D25$_{8bit}$ sub-dataset are used. This is a 3D dataset and so, we employ 3D version of Micro𝕊plit. While the task XXXI works directly on the above-mentioned data whereas task XXXII adds Gaussian and Poisson noise on top of the individual channels. Please refer to Supplementary Note 1.1.1 on how the noise was added. On a technical node, since the task XXXI directly works on the denoised data, there is no rationale to use noise models on such data. Hence the denoi𝕊plit loss component is completely disabled ($w = 0$ in Equation 1) and the Task XXXI is effectively trained with μSplit configuration. We show one qualitative example for each task in Extended Data Figure 4.

Task XXXIII-XXXV

These three tasks are 3-channel 3D semantic unmixing tasks generated from HHMI-D25$_{16bit}$ dataset. Mitochondria, lysosomes and nuclei channels from HHMI-D25$_{16bit}$ sub-dataset are used. This is a 3D dataset and so, we employ 3D version of Micro𝕊plit. While the task XXXIII works directly on the above-mentioned data whereas tasks XXXIV and XXXV adds Gaussian and Poisson noise on top of the individual channels. Please refer to Supplementary Note 1.1.1 on how the noise was added. We show one qualitative example for each task in Extended Data Figure 3.

Task XXXVI

This task is a 3-channel 3D semantic unmixing task generated from HHMI-D25$_{16bit,0.25}$ dataset. Mitochondria, lysosomes and nuclei channels from HHMI-D25$_{16bit,0.25}$ sub-dataset are used. This is a 3D dataset and so, we employ 3D version of Micro𝕊plit. We show one qualitative example for the task in Supplementary Figure 54.

4.3 Four-channel semantic unmixing tasks

Task XXIX

This task is generated from the HT-LIF24 dataset and uses all four structures to create this task. The exposure duration used for this task is $5ms$. We show one qualitative example per task in Figure 1f.

Task XXX

This task is generated from the Chicago-Sch23 dataset and uses all four structures to create this task. Due to the same reason as described for Tasks `XV-XX`, we have disabled the Noise model for this task as well. We show one qualitative example in Supplementary Figure 5.

	GT Noisy vs GT High-SNR						Prediction vs GT High-SNR					
Acq.	PSNR			MicroMS-SSIM			PSNR			MicroMS-SSIM		
Duration	C1	C2	C3	C1	C2	C3	C1	C2	C3	C1	C2	C3
2ms	23.3	25.1	26.1	0.839	0.772	0.869	31.0	32.2	36.3	0.940	0.973	0.944
3ms	23.6	26.0	27.2	0.842	0.780	0.871	30.8	32.2	36.1	0.940	0.973	0.948
5ms	24.6	28.3	29.9	0.857	0.817	0.875	32.9	34.3	37.6	0.960	0.983	0.963
20ms	30.0	35.6	38.16	0.920	0.942	0.914	37.0	39.7	41.4	0.984	0.994	0.989

Table 11 Photon efficient imaging with MicroSplit: a concrete example. Denoising enables repurposing of the available photon budget by acquiring lower-SNR micrographs, which MicroSplit can restore to high-SNR predictions. We compare raw data acquired at 2ms, 3ms, 5ms, and 20ms exposure times with high-SNR (500ms) reference images of the same regions in the HT-LIF24 dataset (three channels: Nucleus, Microtubules, Kinetochore). The first six data columns in the table quantify the similarity of low-exposure raw data to the 500ms reference, while the rightmost six columns show the corresponding similarities for MicroSplit predictions (identical data as in Table1). In all cases, the MicroSplit predictions exhibit higher quality than the corresponding raw inputs. Strikingly, predictions from 2ms exposures already surpass the quality of 5ms raw data for all channels, and for Channel 1 even exceed the 20ms raw data. In this example, this corresponds to at least a three-fold reduction in required photon budget per acquisition, and likely closer to an order of magnitude when averaged across the three channels.

Task Idx	Dataset	Task Details	2D/3D	N	PSNR		MicroMS-SSIM	
					C1	C2	C1	C2
I	HT-H24	-	3D	15	0.09	0.55	0.973	0.956
II	HT-P23A	-	3D	9	0.53	0.45	0.016	0.006
III	HT-P23B	-	3D	13	0.29	0.39	0.005	0.019
IV	Pavia-P24	high, 50/50	2D	2	0.17	1.1	0.010	0.009
V	Pavia-P24	high, 66/33	2D	2	1.83	0.19	0.045	0.002
VI	Pavia-P24	high, 84/16	2D	2	0.35	1.44	0.016	0.049
VII	Pavia-P24	mid, 50/50	2D	2	0.26	0.55	0.005	0.013
VIII	Pavia-P24	mid, 66/33	2D	2	0.25	0.09	0.009	0.003
IX	Pavia-P24	mid,84/16	2D	2	0.02	0.42	0.005	0.006
X	Pavia-P24	low, 50/50	2D	2	0.41	0.42	0.010	0.012
XI	Pavia-P24	low, 66/33	2D	2	0.02	0.28	0.006	0.006
XII	Pavia-P24	low, 84/16	2D	2	0.72	0.76	0.025	0.018
XIII	HT-T24	-	2D	36	0.66	0.52	0.005	0.004
XIV	HT-LIF24	-	2D	10	0.66	1.21	0.003	0.004
XV	Chicago-Sch23	C0 vs C1	2D	10	1.80	0.92	0.003	0.001
XVI	Chicago-Sch23	C0 vs C2	2D	10	1.68	1.32	0.005	0.002
XVII	Chicago-Sch23	C0 vs C3	2D	10	1.20	0.54	0.000	0.000
XVIII	Chicago-Sch23	C1 vs C2	2D	10	0.89	1.46	0.001	0.001
XIX	Chicago-Sch23	C1 vs C3	2D	10	0.99	0.81	0.000	0.001
XX	Chicago-Sch23	C2 vs C3	2D	10	1.01	0.82	0.000	0.000

Task Idx	Dataset	Task Details	2D/3D	N	PSNR			MicroMS-SSIM		
					C1	C2	C3	C1	C2	C3
XXI	CBG-Z18	-	3D	69	0.12	0.18	0.12	0.002	0.001	0.001
XXII	CBG-N18	-	3D	30	0.18	0.21	0.06	0.000	0.000	0.000
XXIII	HHMI-D25$_{8bit}$	-	3D	51	0.018	0.035	0.027	0.000	0.002	0.001
XXIV	HT-LIF24	2ms	2D	10	1.19	0.90	0.66	0.007	0.003	0.004
XXV	HT-LIF24	3ms	2D	10	1.30	0.96	0.75	0.008	0.003	0.004
XXVI	HT-LIF24	5ms	2D	10	1.11	0.89	0.53	0.004	0.002	0.004
XXVII	HT-LIF24	20ms	2D	10	0.85	0.62	0.73	0.002	0.001	0.002
XXVIII	HT-LIF24	500ms	2D	10	0.61	0.35	0.66	0.001	0.001	0.001
XXXI	HHMI-D25$_{8bit,denoised}$	-	3D	51	0.073	0.11	0.151	0.000	0.001	0.001
XXXII	HHMI-D25$_{8bit,denoised}$	$\sigma = 20, \lambda = 30$	3D	51	0.076	0.094	0.153	0.000	0.003	0.002
XXXIII	HHMI-D25$_{16bit}$	-	3D	52	0.03	0.126	0.016	0.002	0.003	0.001
XXXIV	HHMI-D25$_{16bit}$	$\sigma = 2K, \lambda = 5K$	3D	52	0.031	0.13	0.014	0.002	0.003	0.001
XXXV	HHMI-D25$_{16bit}$	$\sigma = 4K, \lambda = 10K$	3D	52	0.033	0.133	0.016	0.002	0.004	0.001
XXXVI	HHMI-D25$_{16bit,0.25}$	-	3D	52	0.082	0.103	0.021	0.000	0.001	0.000

Task Idx	Dataset	2D/3D	N	PSNR				MicroMS-SSIM			
				C1	C2	C3	C4	C1	C2	C3	C4
XXIX	HT-LIF24	2D	10	0.78	1.05	0.86	0.53	0.004	0.004	0.002	0.006
XXX	Chicago-Sch23	2D	10	1.57	0.87	1.24	0.65	0.006	0.002	0.003	0.008

Table 12 Standard errors for all entries in Table 1 are computed over the number of frames reported in the 'N' column.

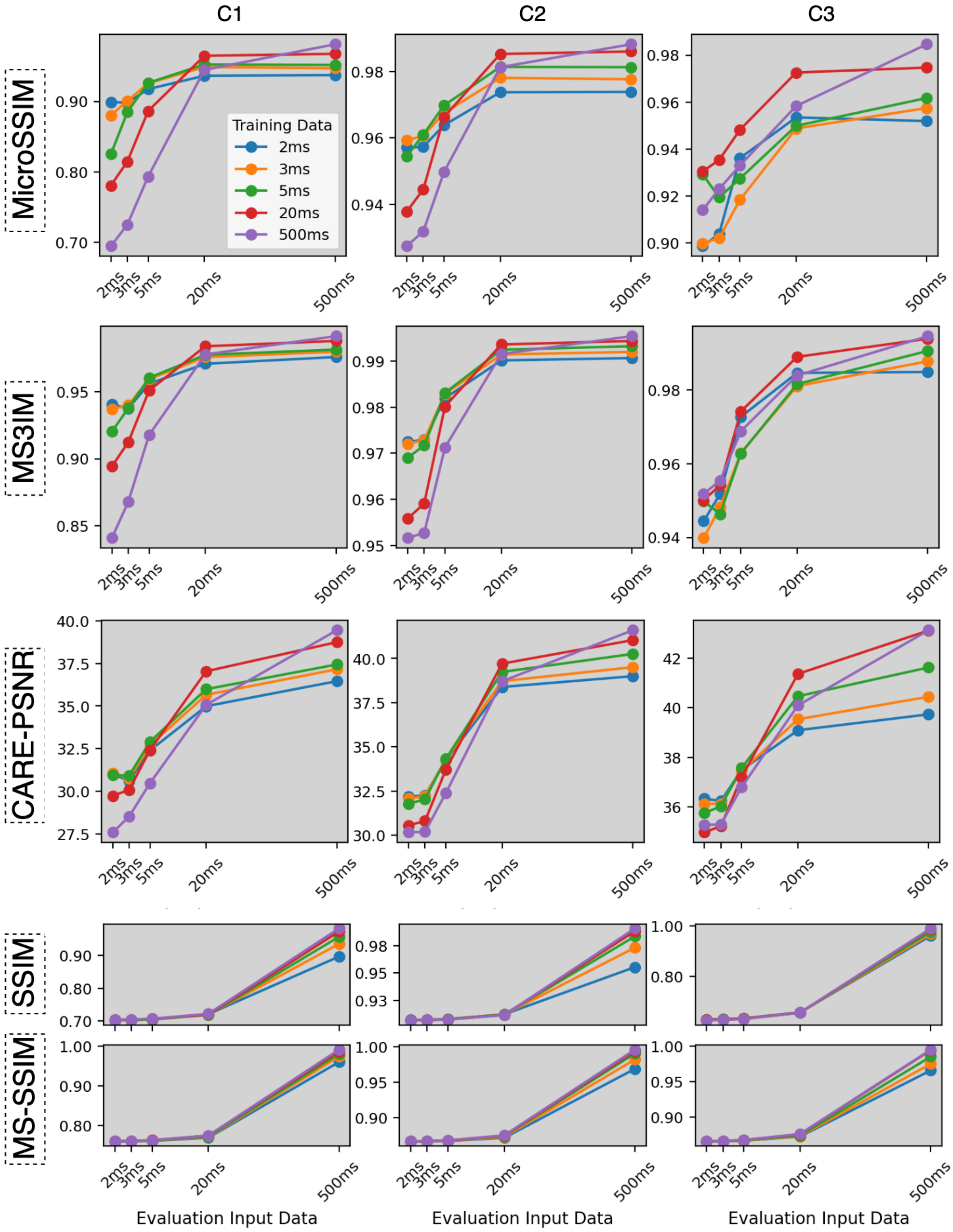

Fig. 1 Quantitative evaluations of the effect of signal-to-noise ratios (SNR) on in-distribution and out-of-distribution unmixing results (using the HT-LIF24 dataset). We trained Micro𝕊plit models on micrographs acquired using a range of different exposure times. Note that the underlying sample ROIs remain identical. We then used all trained models (for 2ms, 3ms, 5ms, 20ms and 500ms exposure time data, indicated in the legend) and evaluated their semantic unmixing performance on all exposure times (x-axis), respectively. The rows show unmixed results quantified using 𝕄icroSSIM, 𝕄icroMS-SSIM, CARE-PSNR, SSIM and MS-SSIM metrics. All plots utilize the legend presented in the plot in the first row, first column. Note that the plots in this figure also demonstrate that the MS-SSIM and SSIM metrics do not work well on microscopy data (while 𝕄icroSSIM and 𝕄icroMS-SSIM show better sensitivity [8]).

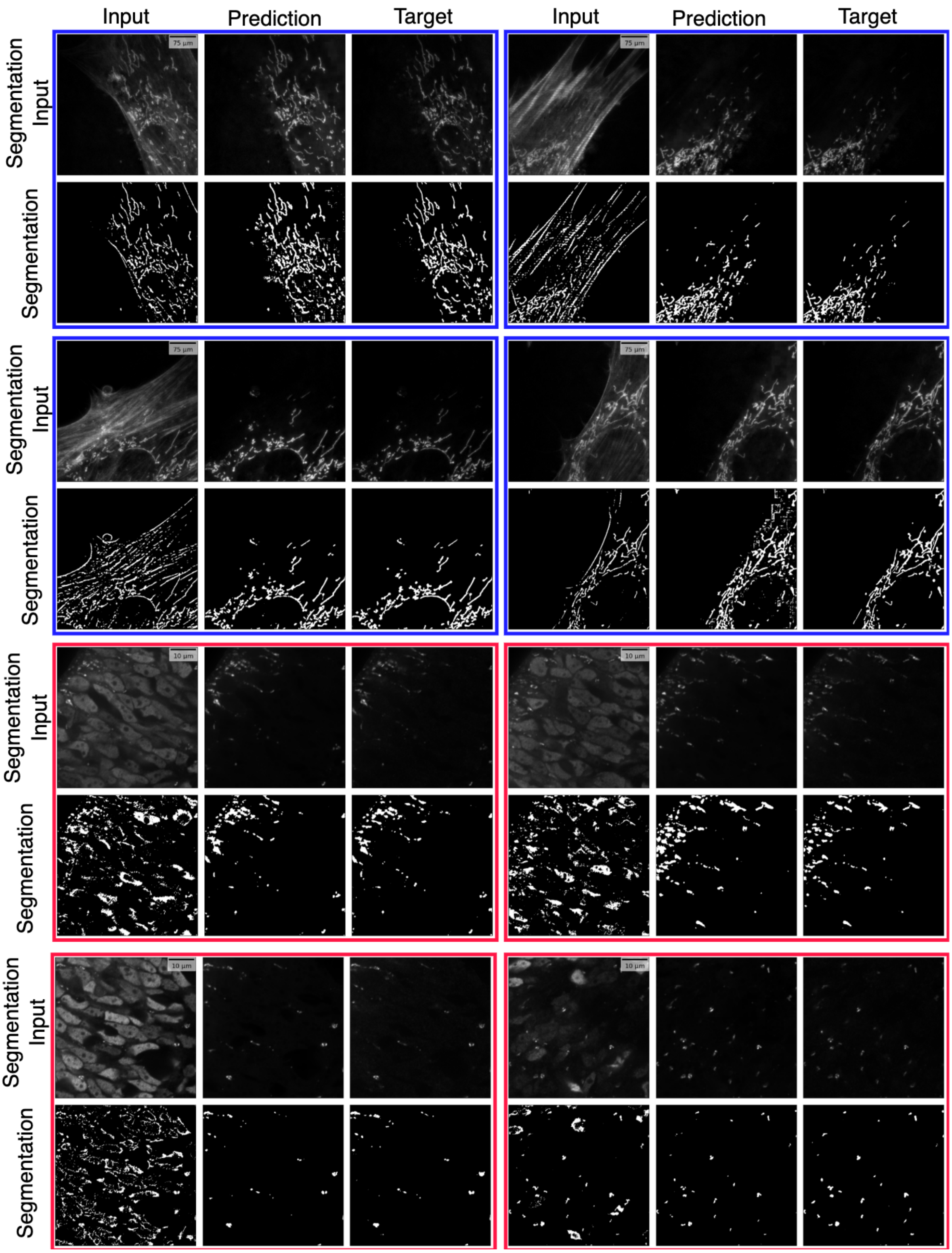

Fig. 2 Segmentation results using common combined labels. In Fig. 3, for training the segmentation model, analysts annotated the target images and trained the segmentation model on target images with those annotations. Similar steps were taken for prediction images. In this experiment, we combined the annotations of both target and prediction images and trained three models, one for target images, one for prediction images, and one for the input images. The purpose for training the segmentation model on the input images was to ascertain whether one can segment one structure from the superimposed input itself. The motivation to use common annotations for training all segmentation models using common annotations was to eliminate the situation where annotation for either target or prediction images were imperfect and hence the segmentation results became inferior to what they should otherwise be.

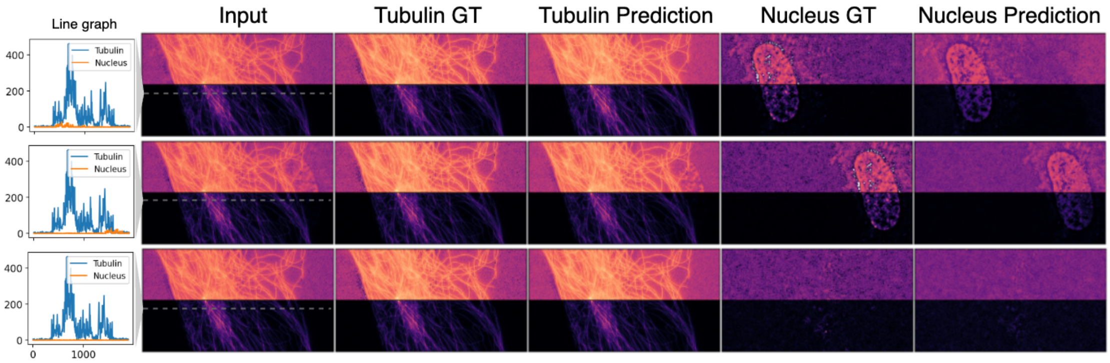

Fig. 3 Unmixing unequally intense channels. Here, we investigate what happens when one of the channels to be unmixed is much dimmer than the other (we called the divergence of structure intensities 'skew' in the main text). We used Tubulin and Nucleus channel from the 4 channel Chicago-Sch23 dataset. Line graphs of Tubulin and Nuclei shows that nucleus channel is considerably dimmer, such that the signal amplitude of this channel is only around the noise amplitude of the Tubulin channel. For every patch, we visualize half of the patch in *LogNorm* so that even the faintest signal becomes visible. We intend to answer the question whether the prediction of nucleus channel is mostly due to learned spatial correlation with the Tubulin channel or if the trained model is indeed capable to unmix such highly skewed intensities. To answer this question, we first trained the Micro𝕊plit network on the original data and then conducted three test-time experiments (showing one per row in the figure). For these experiments, we picked a location in test data which contained both nucleus and tubulin. Micro𝕊plit was training using *Training Mode I*. (*a*) We evaluate on the superimposed image patch created from the pre-selected location. (*b*) We tinkered with the inter-structure spatial correlations by shifting the nucleus channel relative to tubulin channel consistently by 1000 pixels. (*c*) We removed nuclei from the nucleus channel entirely by replacing them with background patches from the same channel. The results clearly indicate that Micro𝕊plit was able to unmix the dim nucleus signals, even if the spatial correlation with the tubulin channel was tinkered with (observe the last two columns, *i.e.* ground truth *vs.* prediction of the nucleus channel).

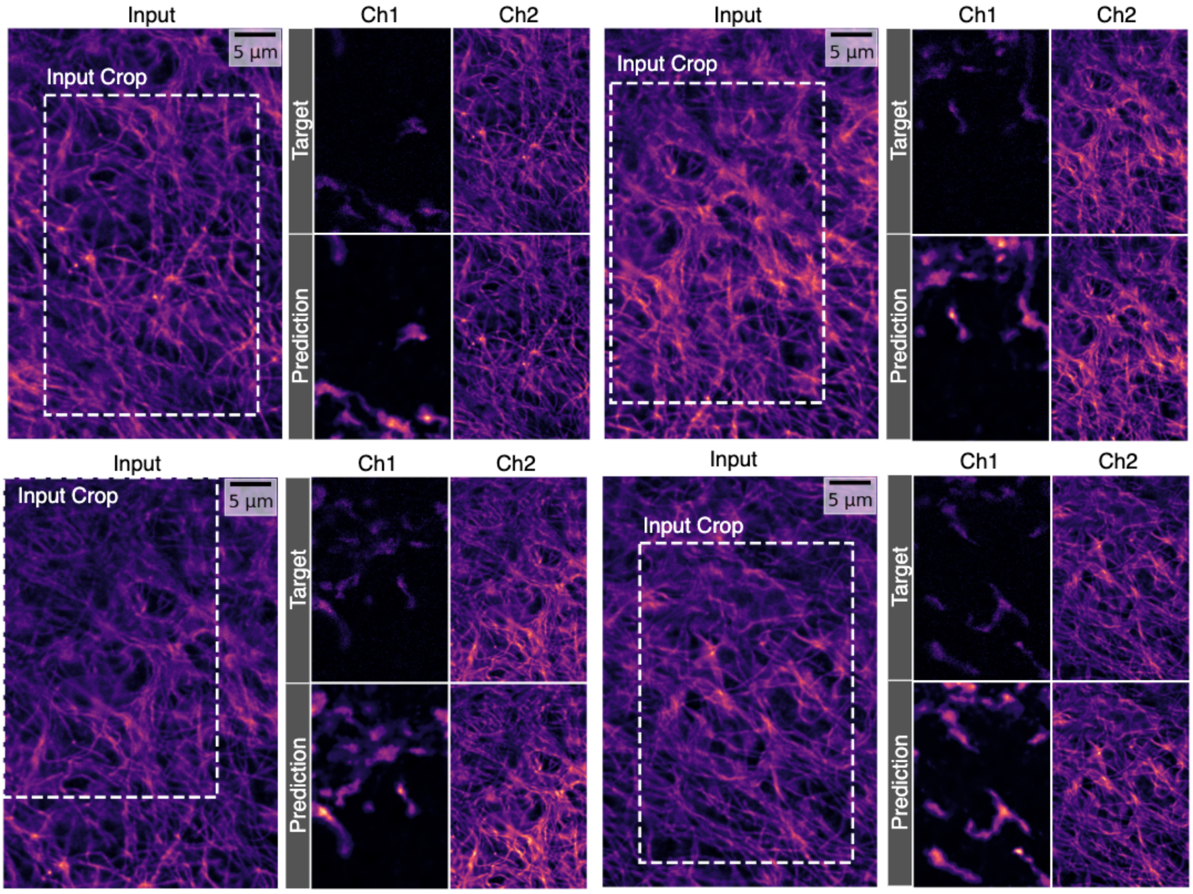

Fig. 4 Task II, HT-P23A This task has a high skew with Mitochondria channel (Ch1) being weaker than Microtubule channel (Ch2), so much so that it is difficult to locate the Mitochondria in the superimposed input. Micro𝕊plit MMSE predictions are subject to a certain 'blurryness' which indicates the model's inability to give a consistent prediction for the Mitochondria channel.

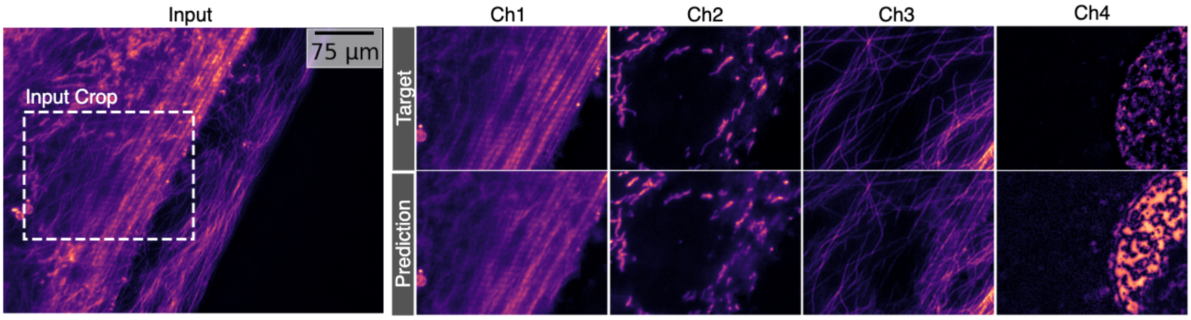

Fig. 5 Qualitative Evaluation for Task XXX from Chicago-Sch23 dataset. Note that we show the target and the prediction corresponding to the input crop which is denoted in *Input* panel by a white dotted rectangle.

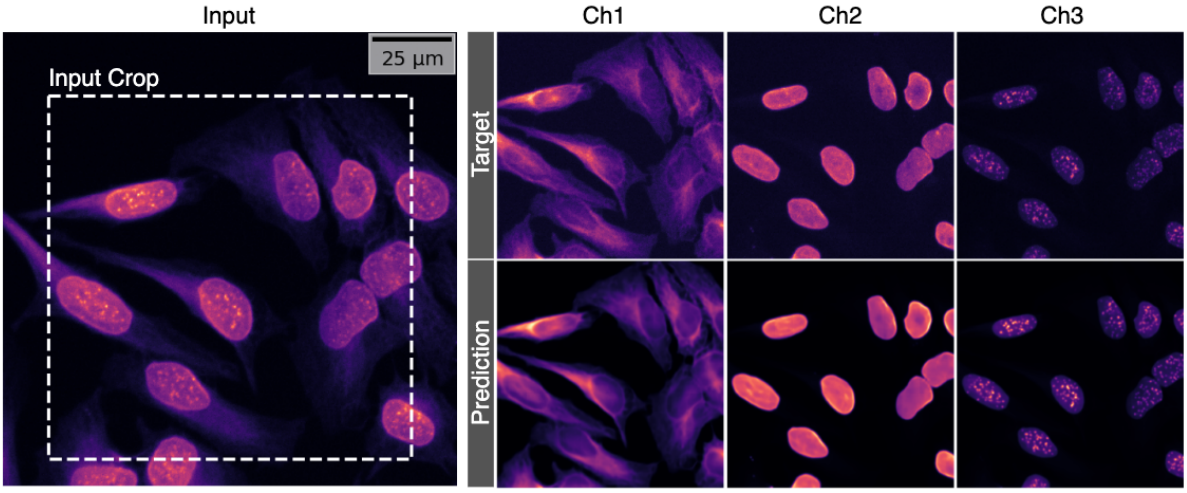

Fig. 6 Qualitative Evaluation for Task XXVII from HT-LIF24 dataset. Note that we show the target and the prediction corresponding to the input crop which is denoted in *Input* panel by a white dotted rectangle.

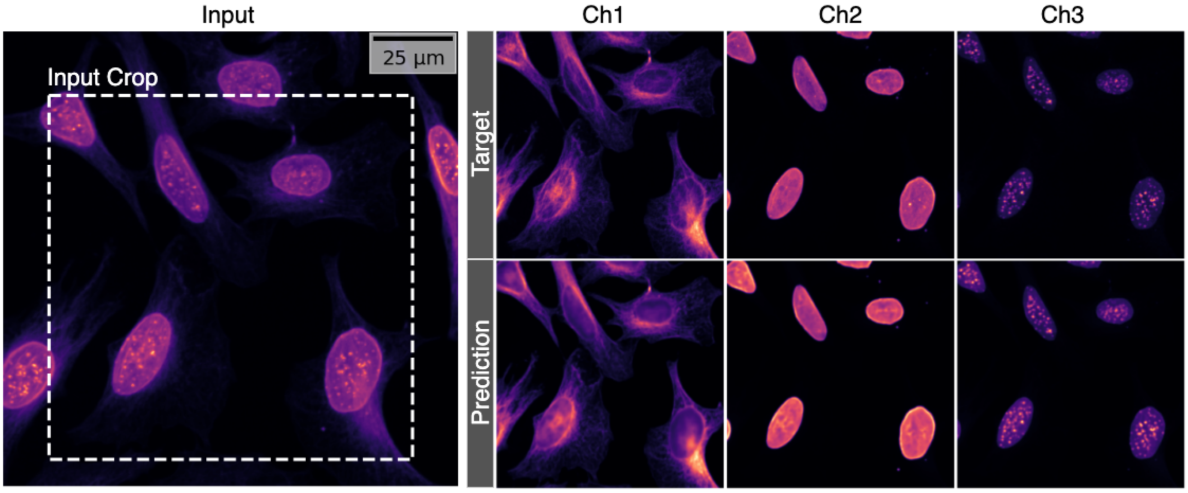

Fig. 7 Qualitative Evaluation for Task XXVIII from HT-LIF24 dataset. Note that we show the target and the prediction corresponding to the input crop which is denoted in *Input* panel by a white dotted rectangle.

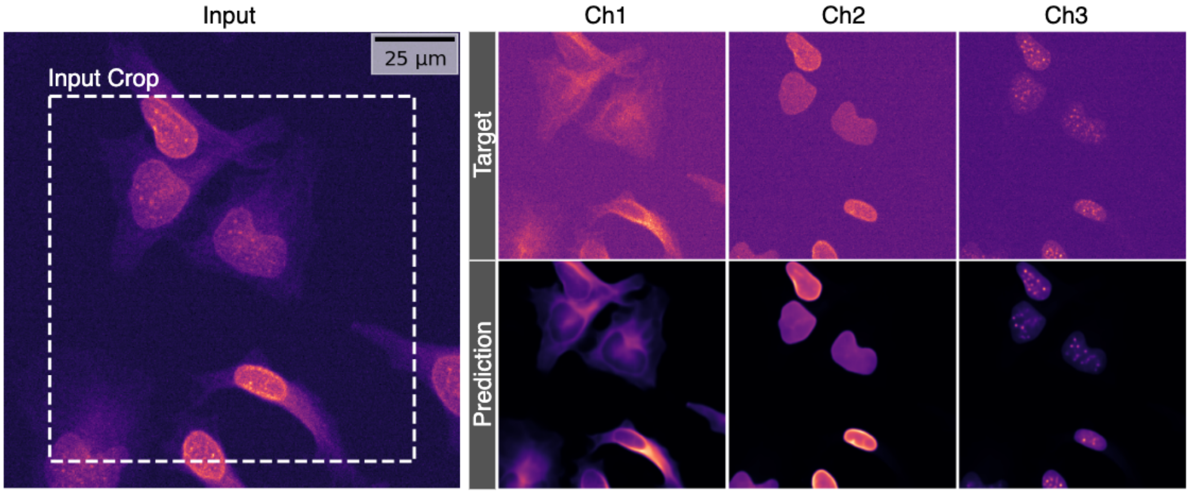

Fig. 8 Qualitative Evaluation for Task XXIV from HT-LIF24 dataset. Note that we show the target and the prediction corresponding to the input crop which is denoted in *Input* panel by a white dotted rectangle.

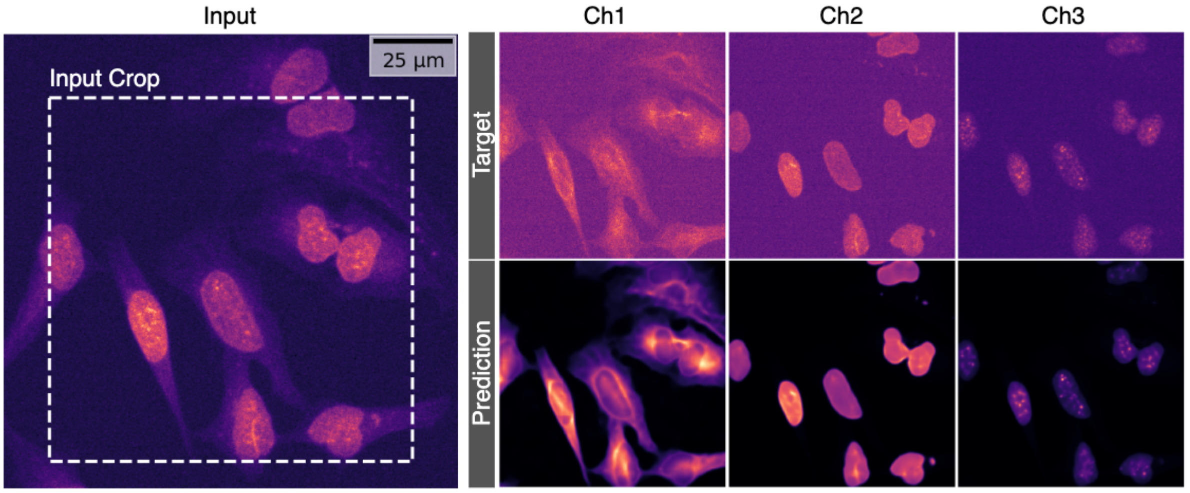

Fig. 9 Qualitative Evaluation for Task XXV from HT-LIF24 dataset. Note that we show the target and the prediction corresponding to the input crop which is denoted in *Input* panel by a white dotted rectangle.

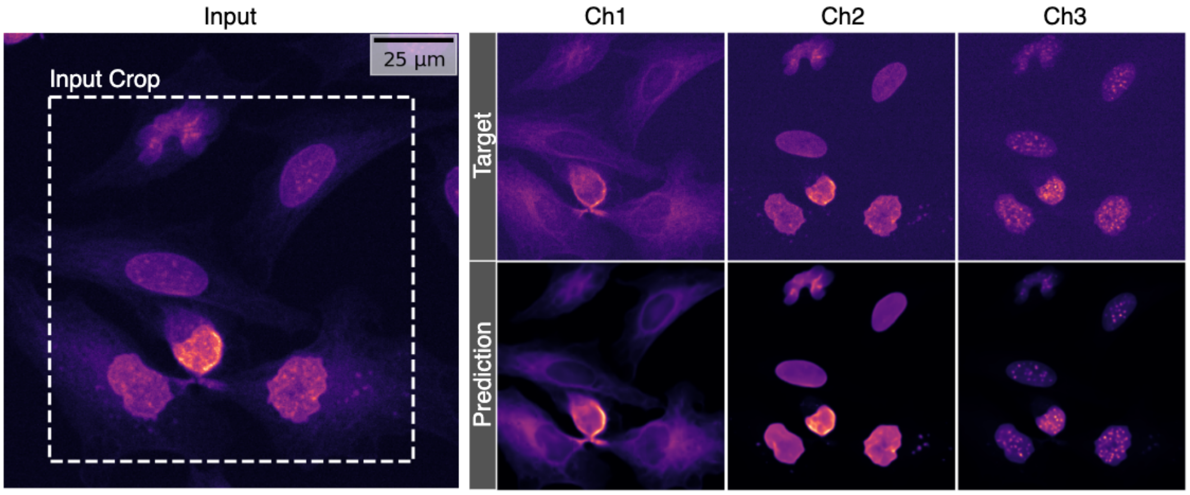

Fig. 10 Qualitative Evaluation for Task XXVI from HT-LIF24 dataset. Note that we show the target and the prediction corresponding to the input crop which is denoted in *Input* panel by a white dotted rectangle.

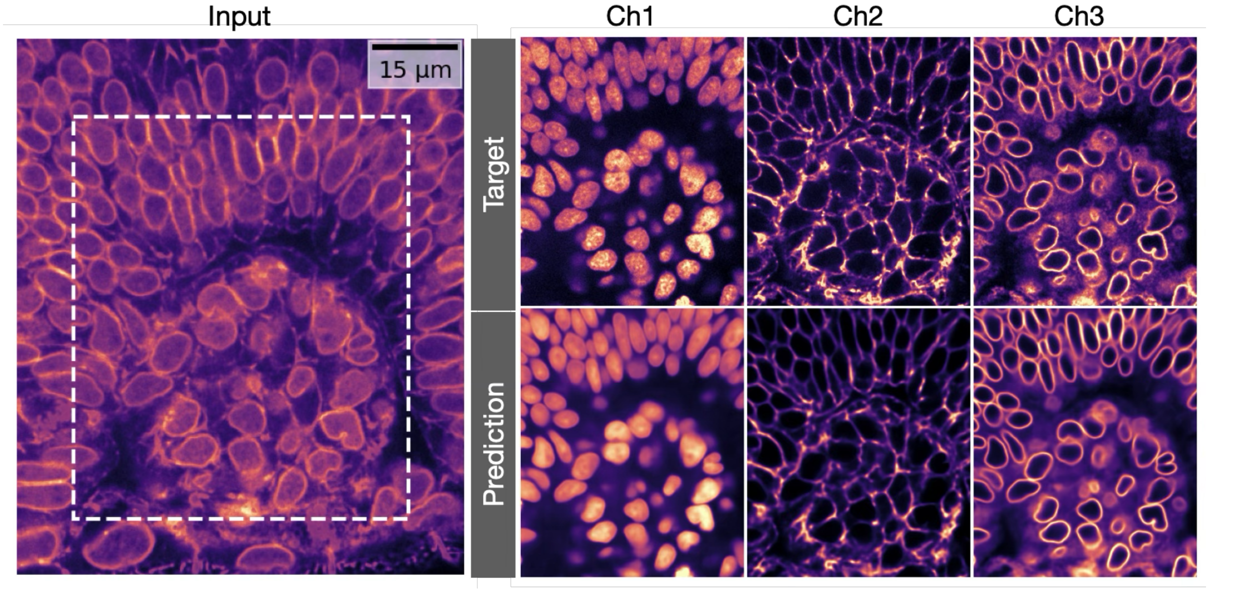

Fig. 11 Qualitative Evaluation for Task XXI from CBG-Z18 dataset. Note that we show the target and the prediction corresponding to the input crop which is denoted in *Input* panel by a white dotted rectangle.

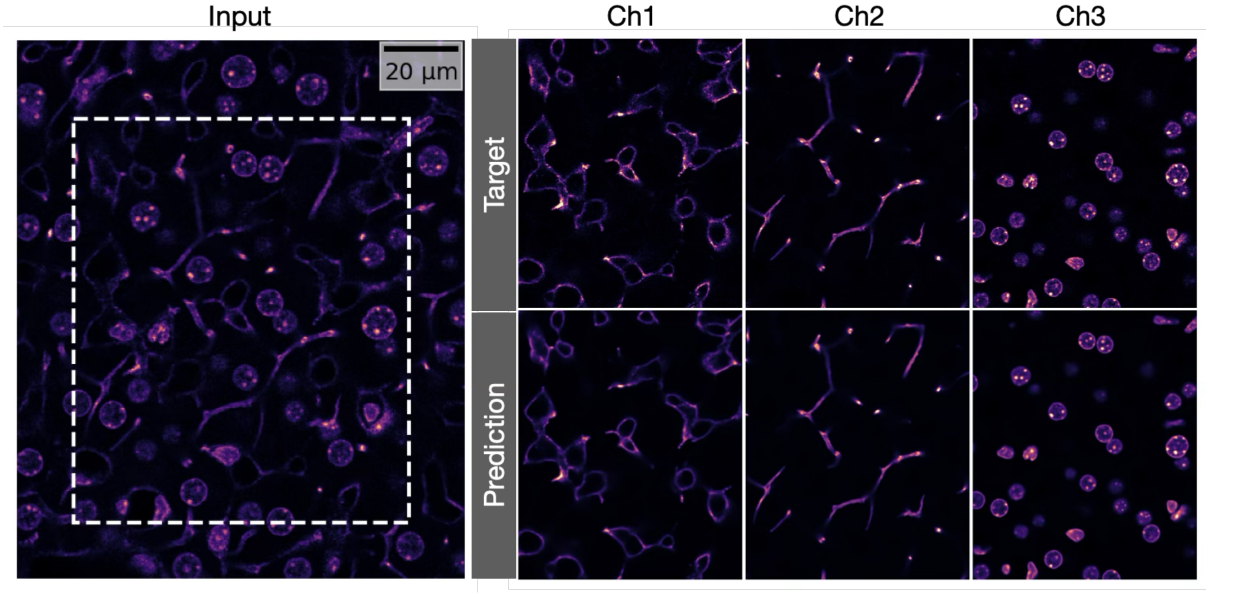

Fig. 12 Qualitative Evaluation for Task XXII from CBG-N18 dataset. Note that we show the target and the prediction corresponding to the input crop which is denoted in *Input* panel by a white dotted rectangle.

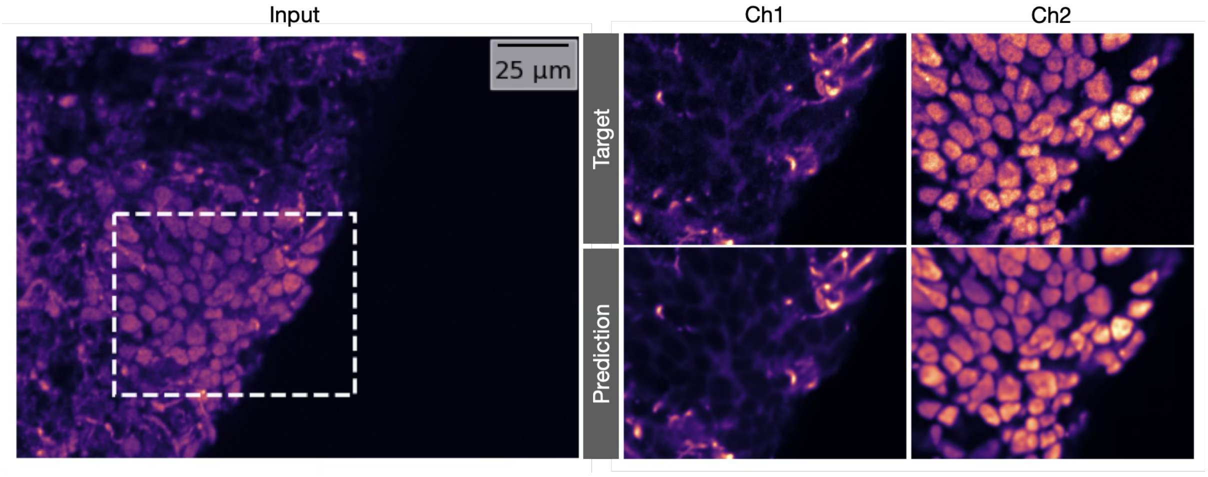

Fig. 13 Qualitative Evaluation for Task I from HT-H24 dataset. Note that we show the target and the prediction corresponding to the input crop which is denoted in *Input* panel by a white dotted rectangle.

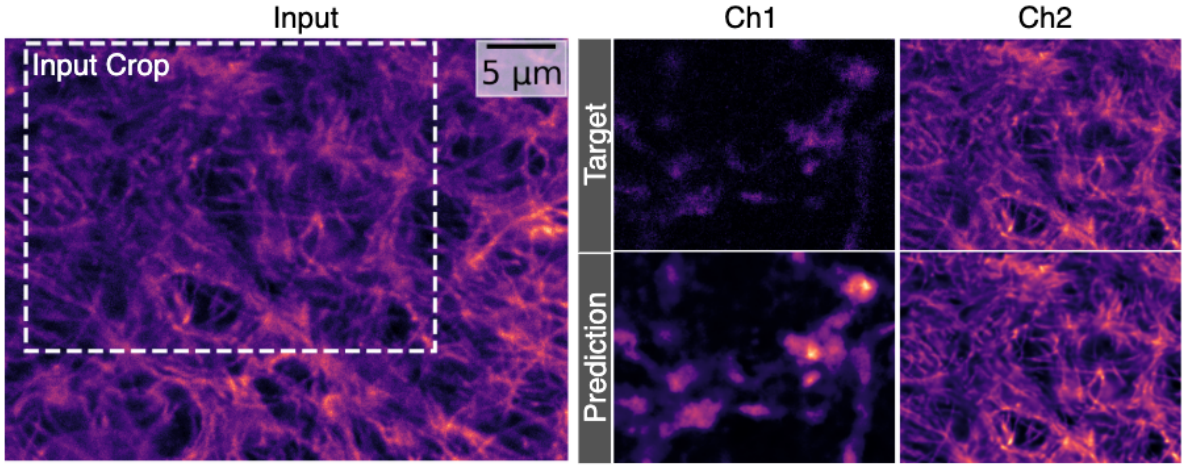

Fig. 14 Qualitative Evaluation for Task II from HT-P23A dataset. Note that we show the target and the prediction corresponding to the input crop which is denoted in *Input* panel by a white dotted rectangle.

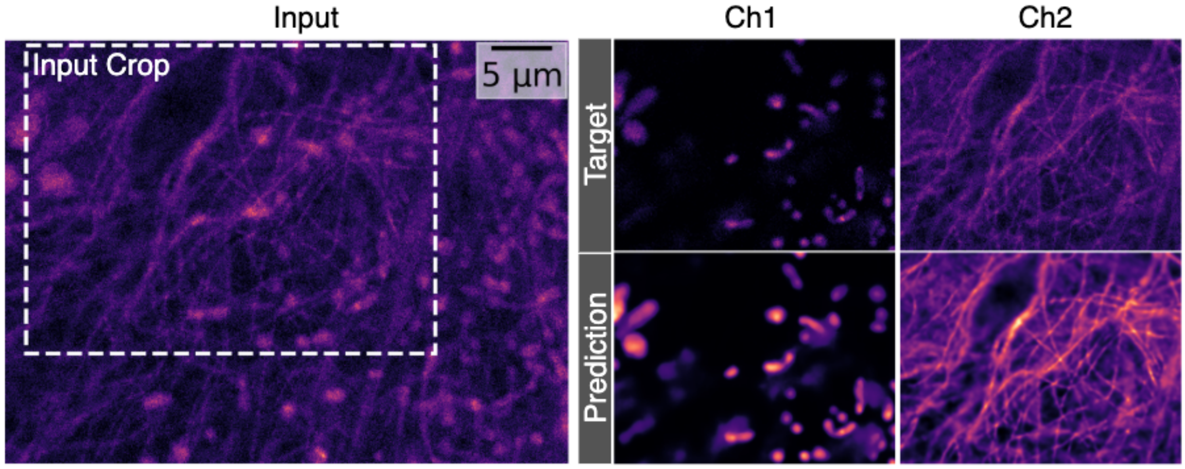

Fig. 15 Qualitative Evaluation for Task III from HT-P23B dataset. Note that we show the target and the prediction corresponding to the input crop which is denoted in *Input* panel by a white dotted rectangle.

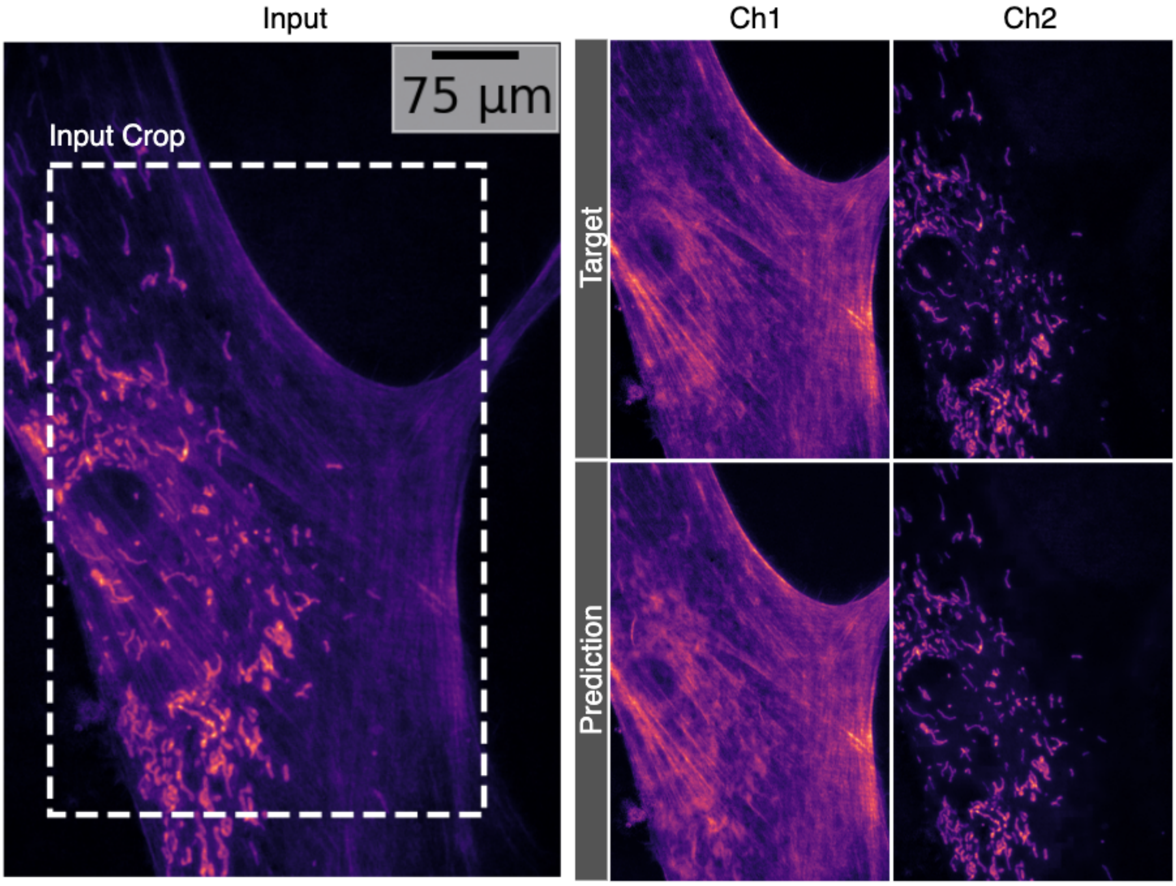

Fig. 16 Qualitative Evaluation for Task XV from Chicago-Sch23 dataset. Note that we show the target and the prediction corresponding to the input crop which is denoted in *Input* panel by a white dotted rectangle.

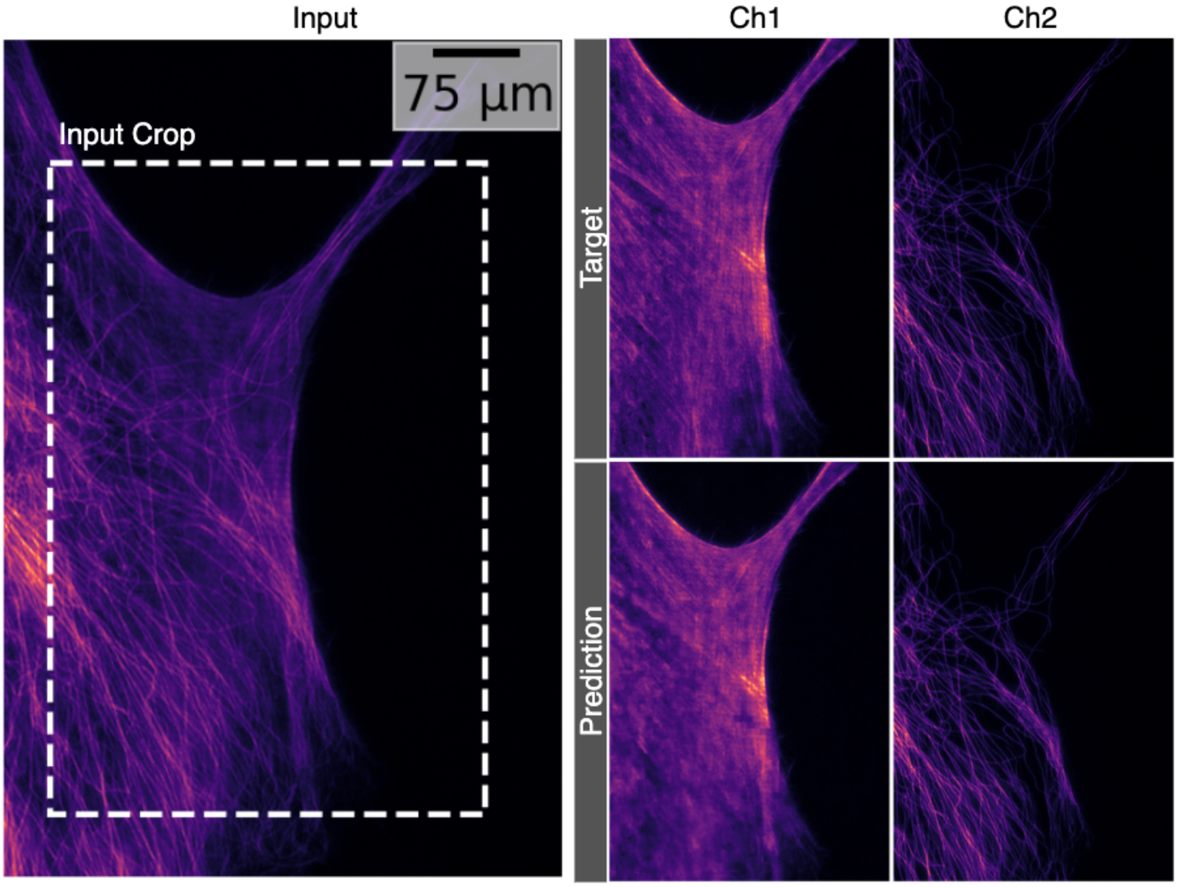

Fig. 17 Qualitative Evaluation for Task XVI from Chicago-Sch23 dataset. Note that we show the target and the prediction corresponding to the input crop which is denoted in *Input* panel by a white dotted rectangle.

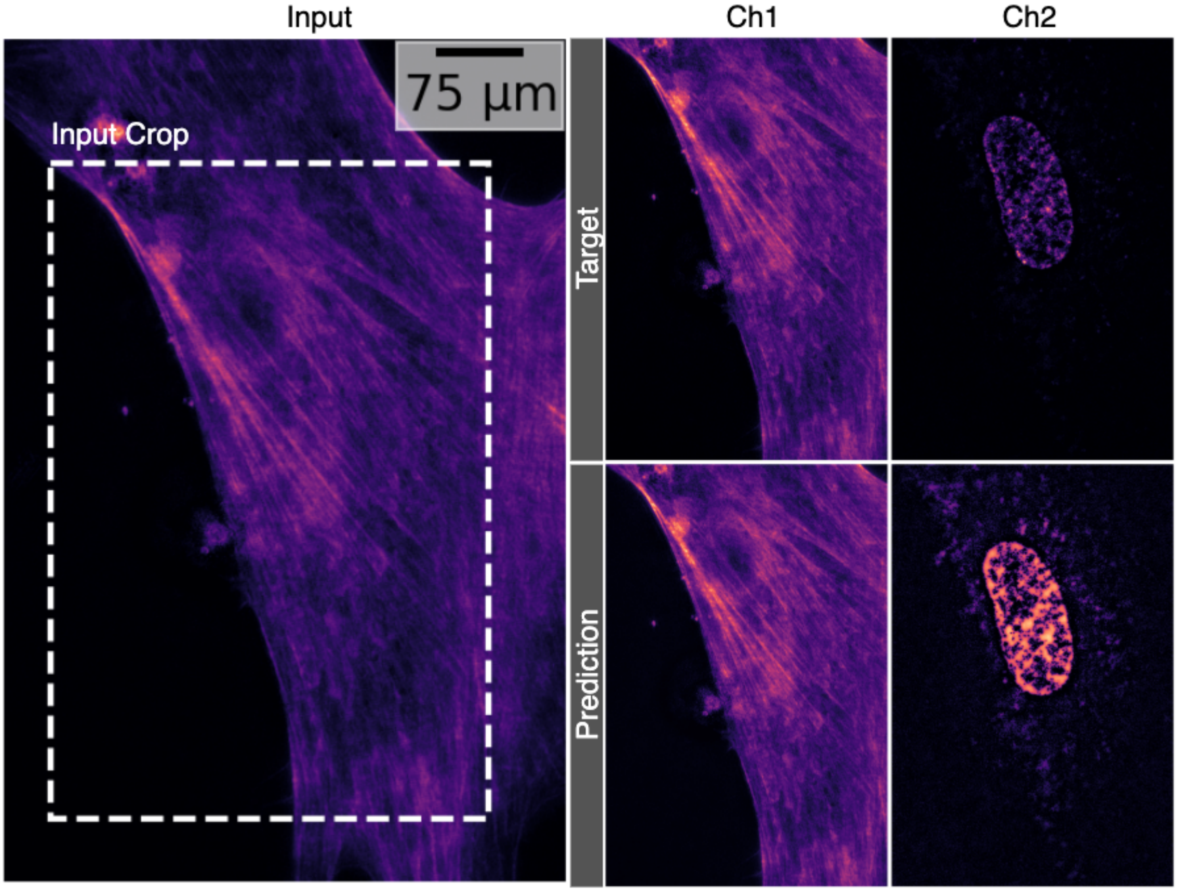

Fig. 18 Qualitative Evaluation for Task XVII from Chicago-Sch23 dataset. Note that we show the target and the prediction corresponding to the input crop which is denoted in *Input* panel by a white dotted rectangle.

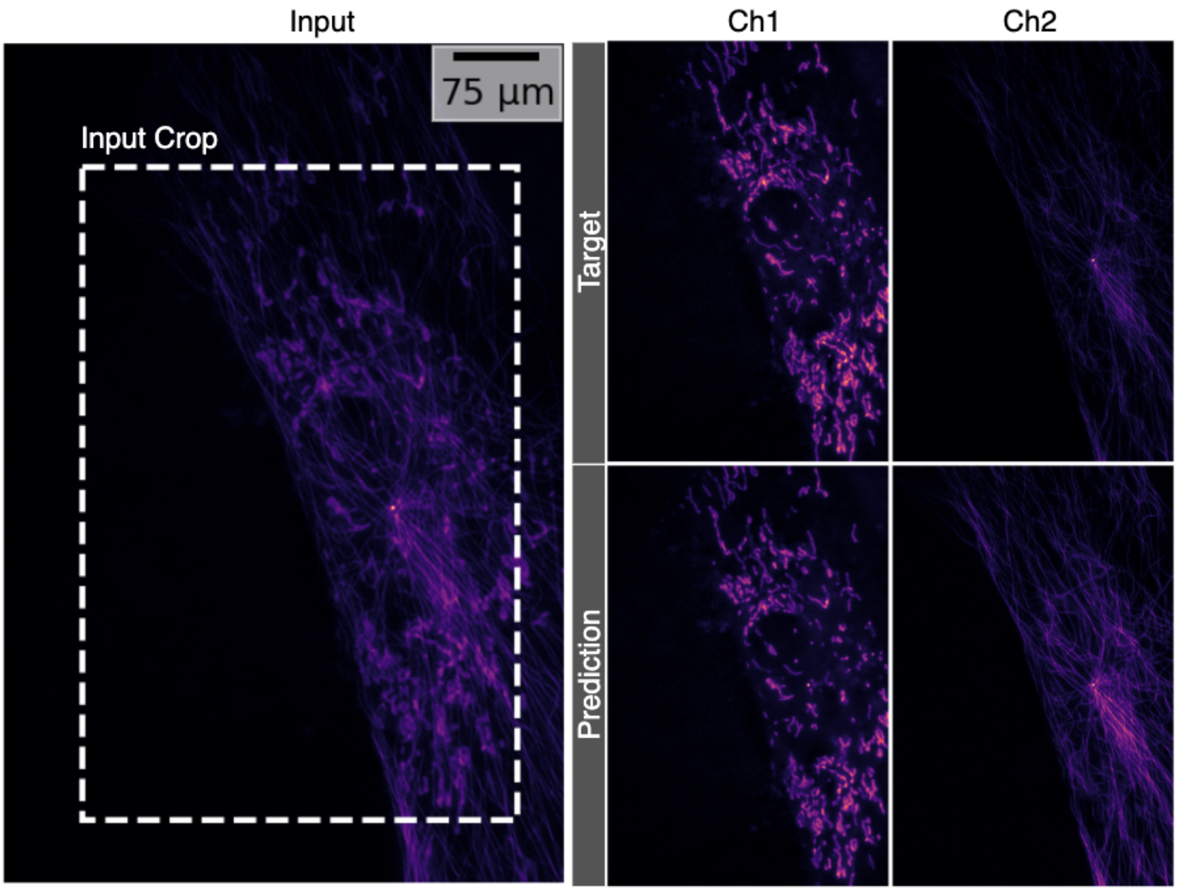

Fig. 19 Qualitative Evaluation for Task XVIII from Chicago-Sch23 dataset. Note that we show the target and the prediction corresponding to the input crop which is denoted in *Input* panel by a white dotted rectangle.

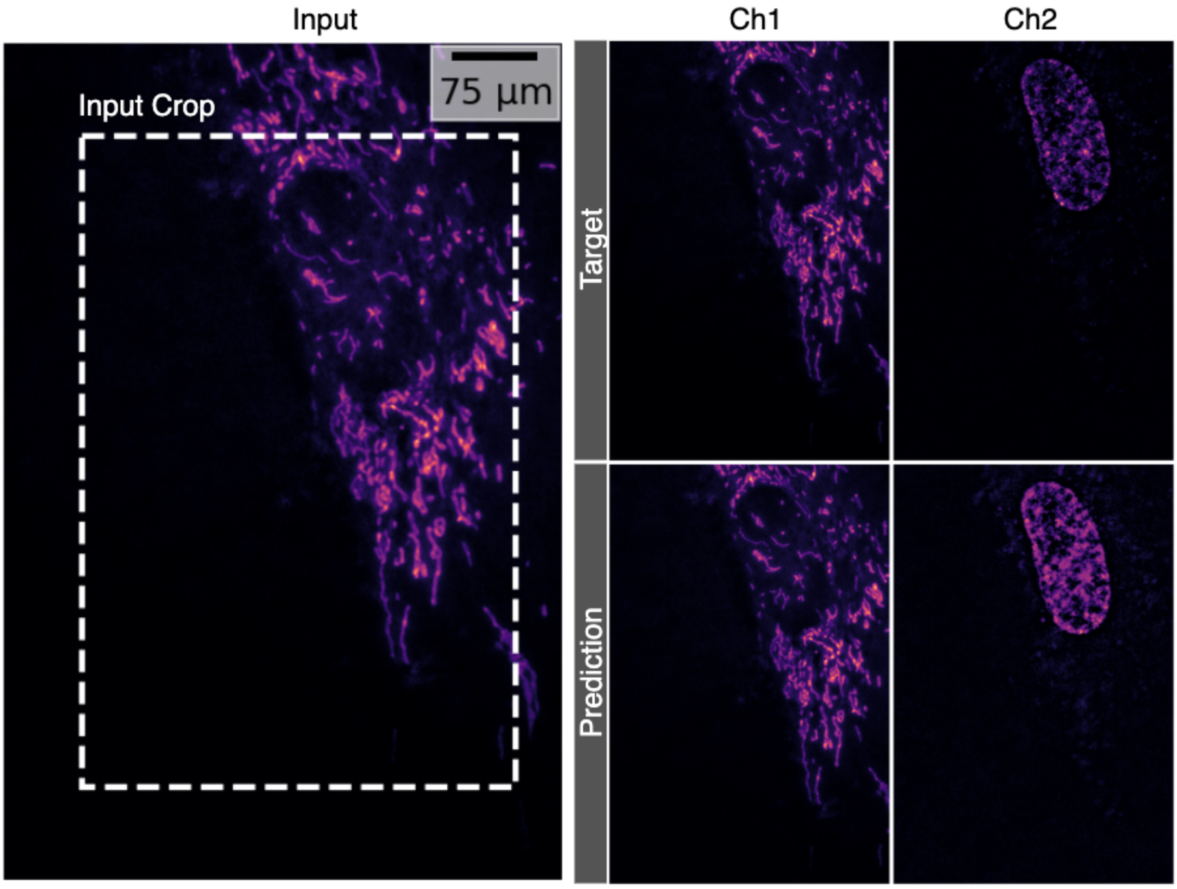

Fig. 20 Qualitative Evaluation for Task XIX from Chicago-Sch23 dataset. Note that we show the target and the prediction corresponding to the input crop which is denoted in *Input* panel by a white dotted rectangle.

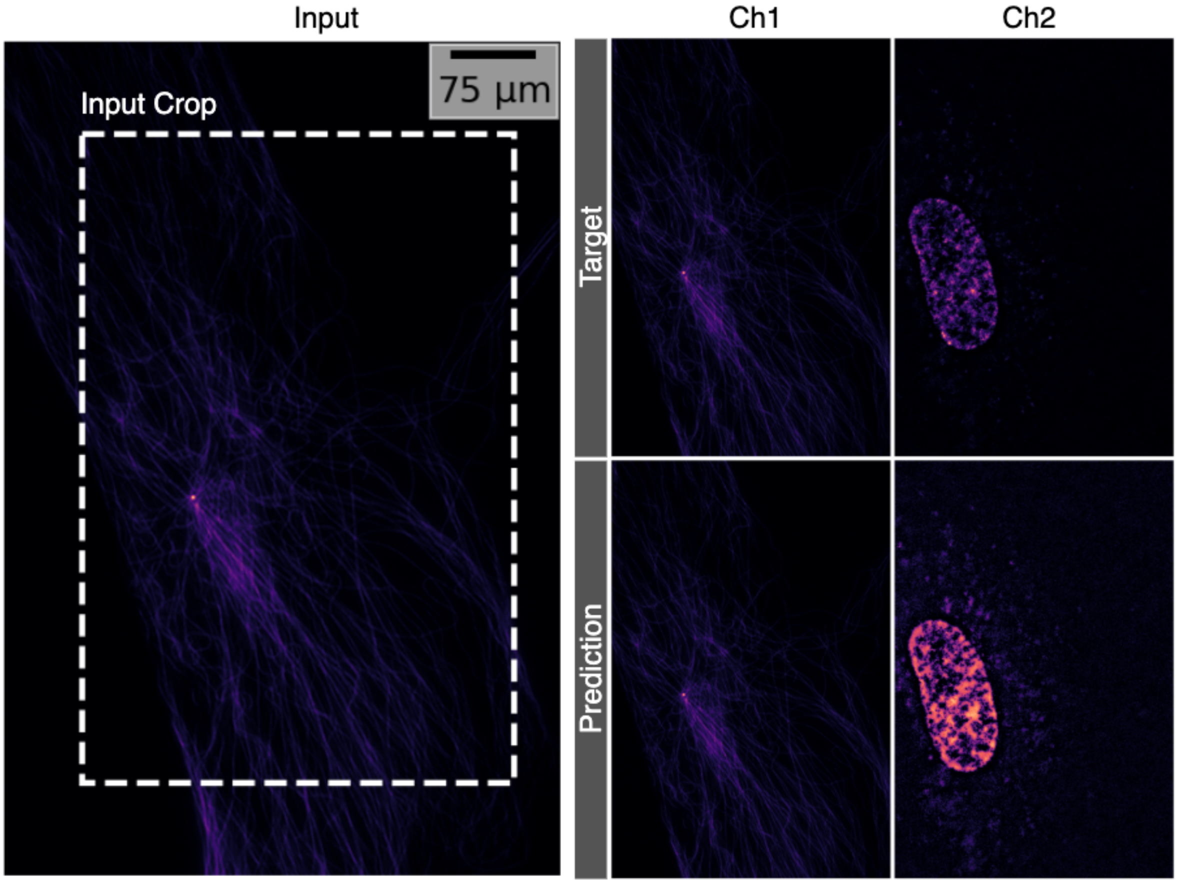

Fig. 21 Qualitative Evaluation for Task XX from Chicago-Sch23 dataset. Note that we show the target and the prediction corresponding to the input crop which is denoted in *Input* panel by a white dotted rectangle.

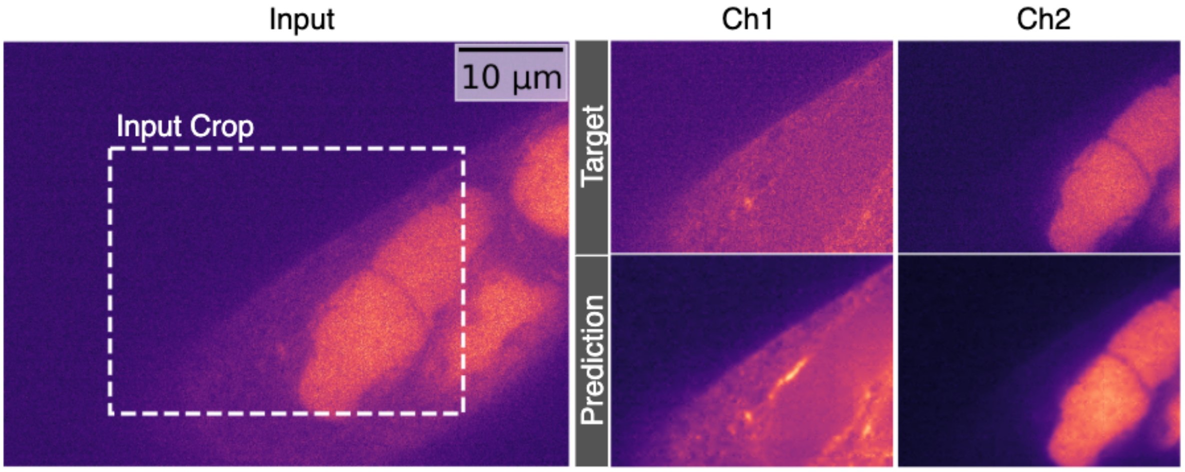

Fig. 22 Qualitative Evaluation for Task IV from Pavia-P24 dataset. Note that we show the target and the prediction corresponding to the input crop which is denoted in *Input* panel by a white dotted rectangle.

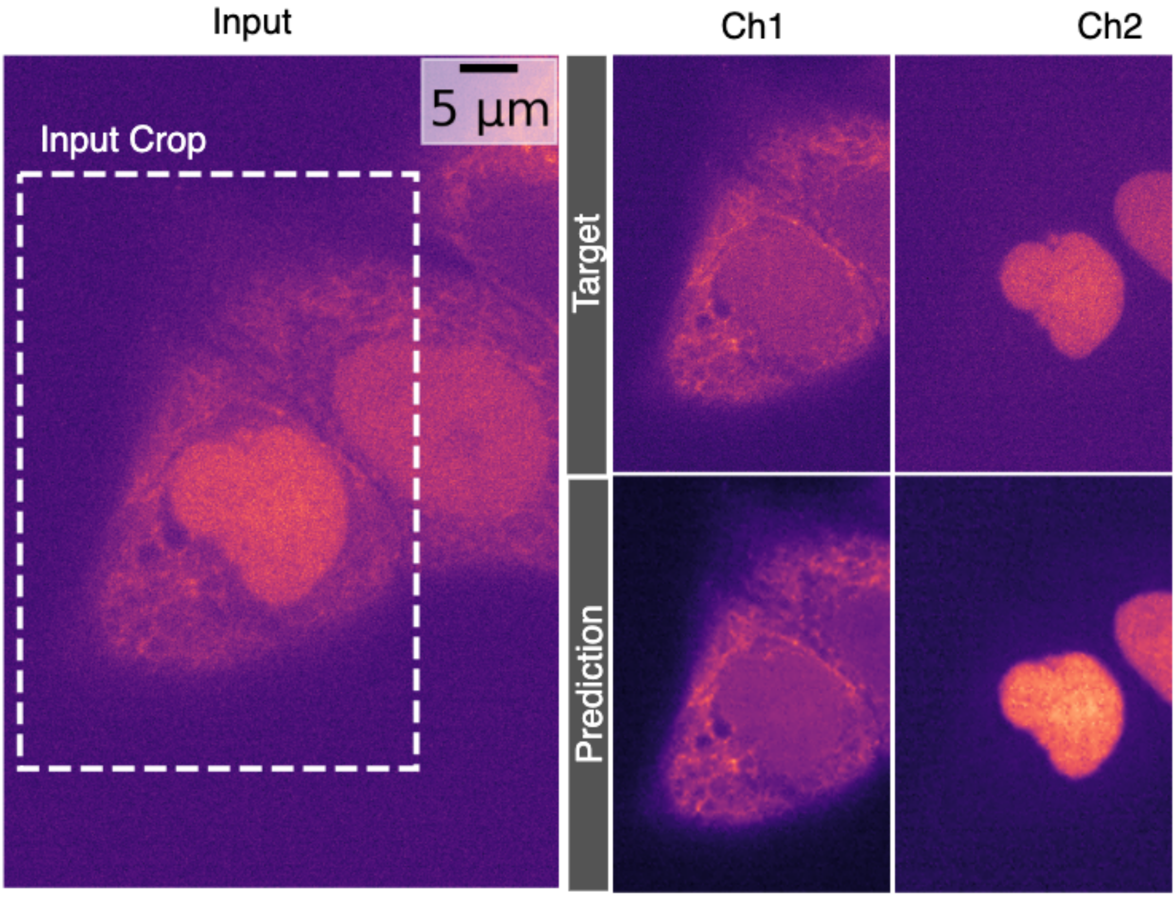

Fig. 23 Qualitative Evaluation for Task VI from Pavia-P24 dataset. Note that we show the target and the prediction corresponding to the input crop which is denoted in *Input* panel by a white dotted rectangle.

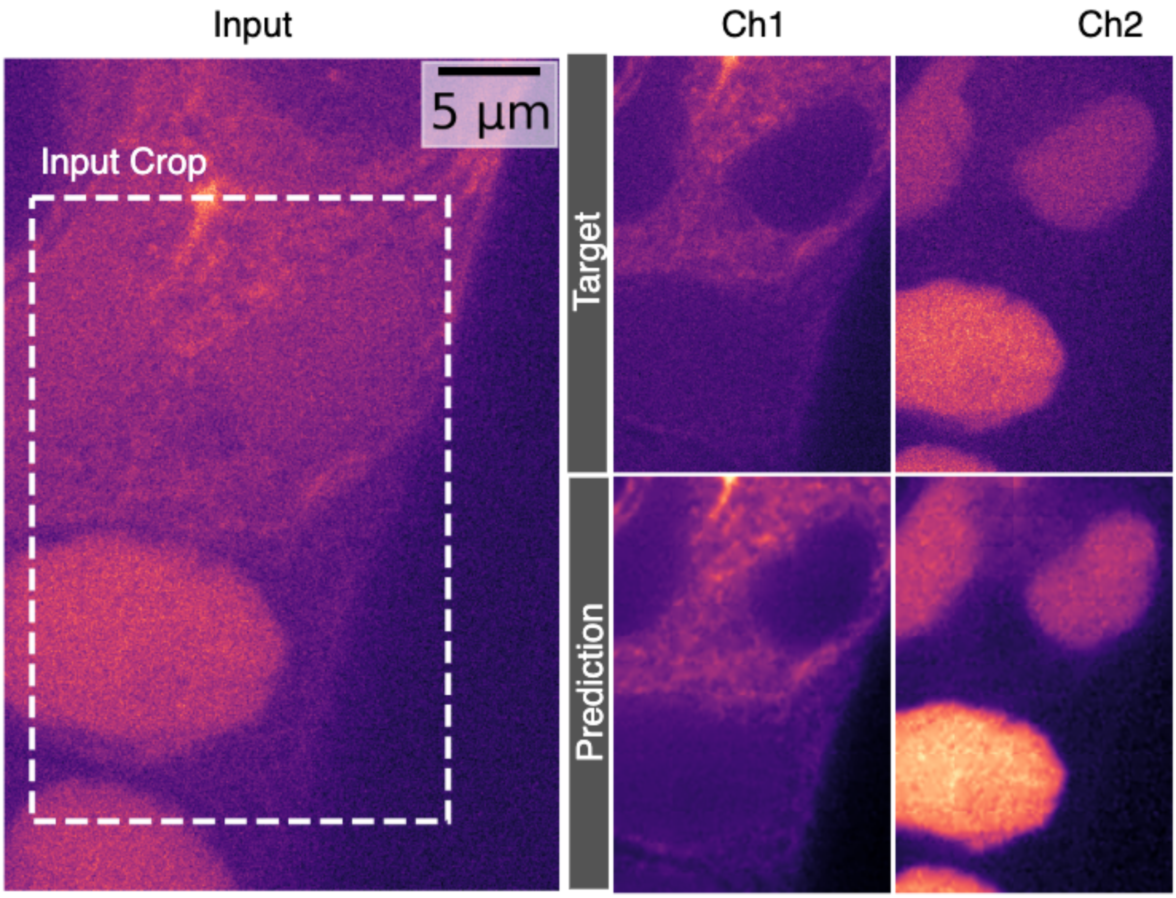

Fig. 24 Qualitative Evaluation for Task V from Pavia-P24 dataset. Note that we show the target and the prediction corresponding to the input crop which is denoted in *Input* panel by a white dotted rectangle.

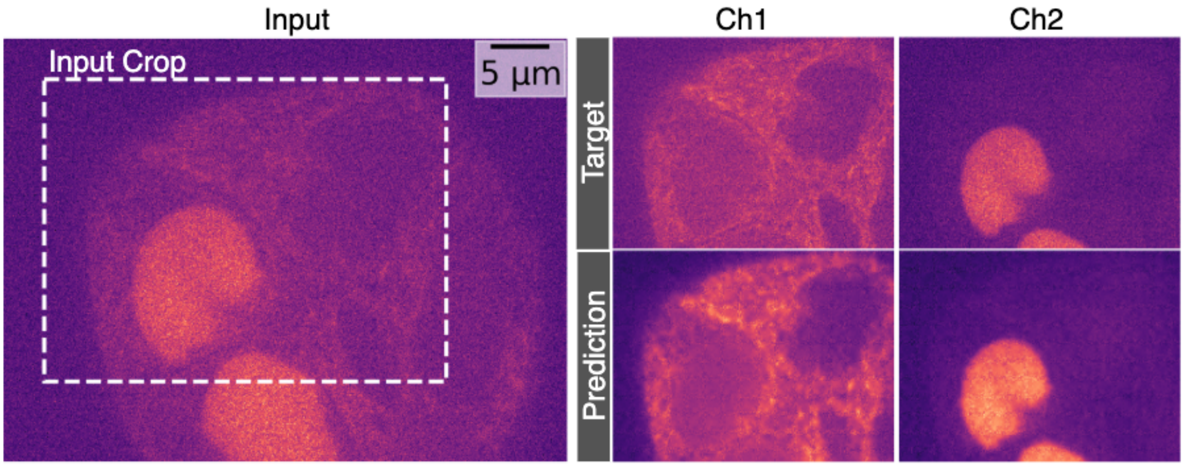

Fig. 25 Qualitative Evaluation for Task VII from Pavia-P24 dataset. Note that we show the target and the prediction corresponding to the input crop which is denoted in *Input* panel by a white dotted rectangle.

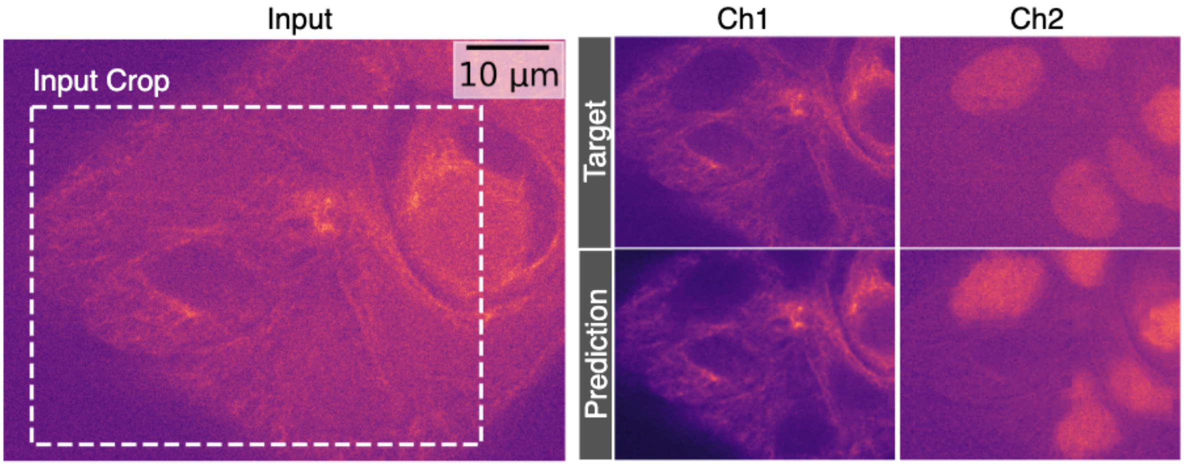

Fig. 26 Qualitative Evaluation for Task VIII from Pavia-P24 dataset. Note that we show the target and the prediction corresponding to the input crop which is denoted in *Input* panel by a white dotted rectangle.

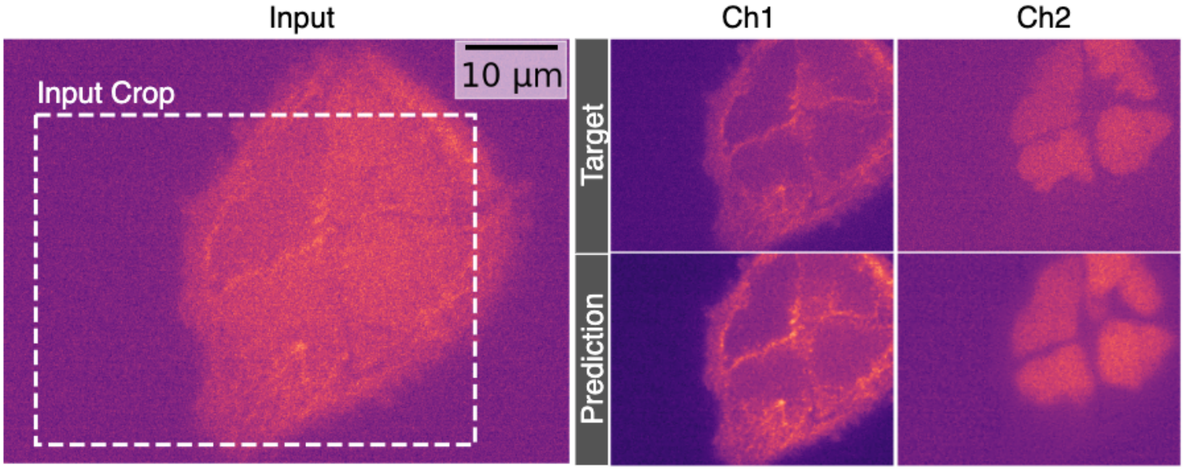

Fig. 27 Qualitative Evaluation for Task IX from Pavia-P24 dataset. Note that we show the target and the prediction corresponding to the input crop which is denoted in *Input* panel by a white dotted rectangle.

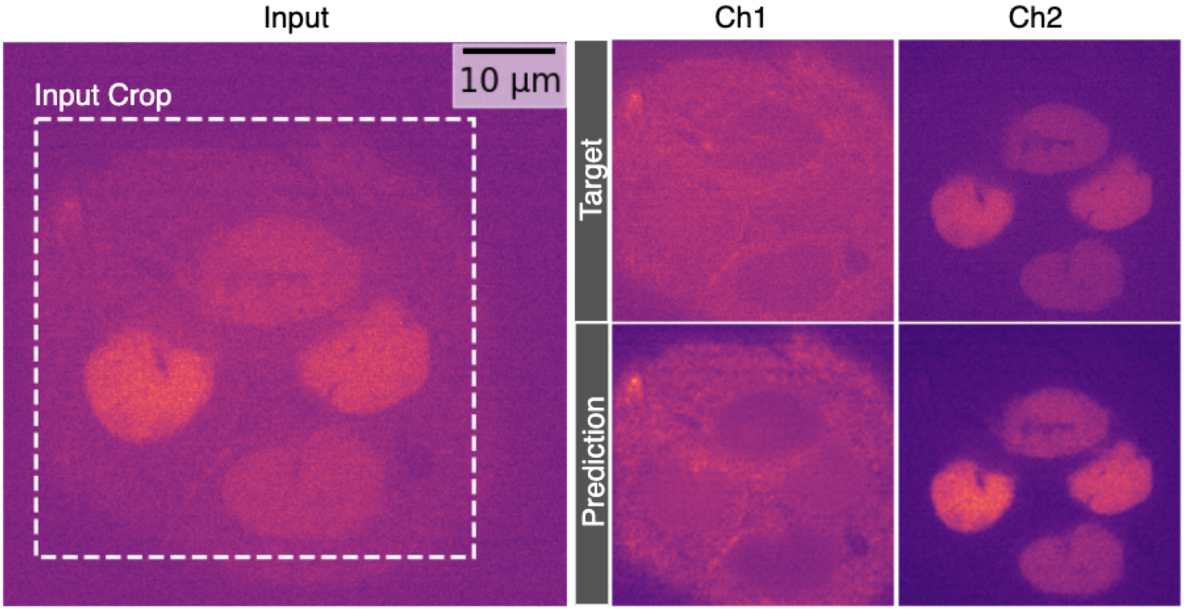

Fig. 28 Qualitative Evaluation for Task X from Pavia-P24 dataset. Note that we show the target and the prediction corresponding to the input crop which is denoted in *Input* panel by a white dotted rectangle.

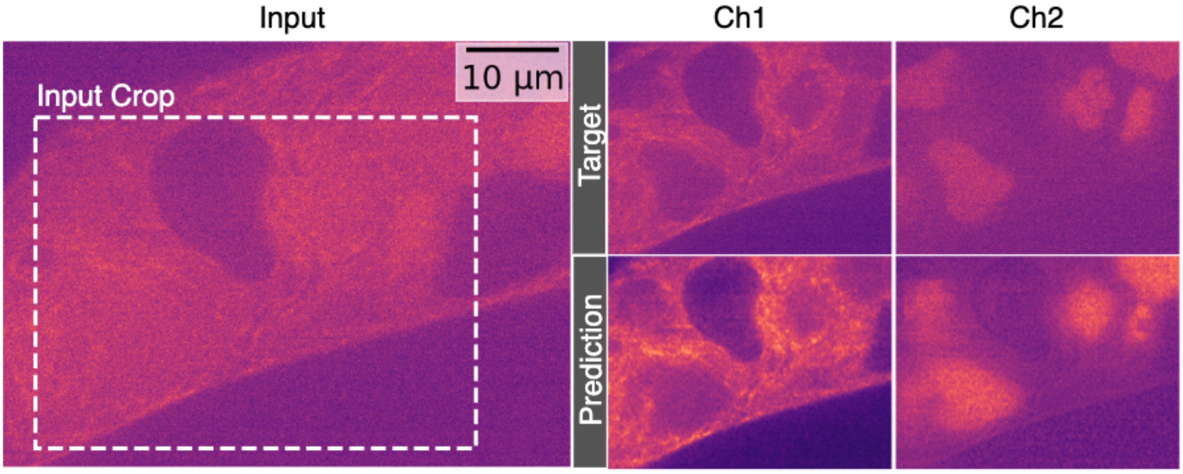

Fig. 29 Qualitative Evaluation for Task XI from Pavia-P24 dataset. Note that we show the target and the prediction corresponding to the input crop which is denoted in *Input* panel by a white dotted rectangle.

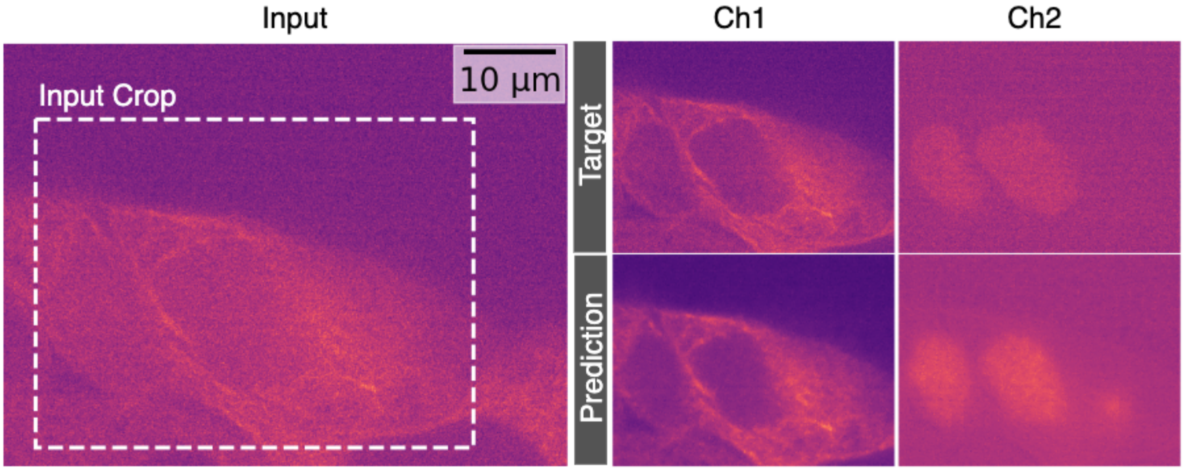

Fig. 30 Qualitative Evaluation for Task XII from Pavia-P24 dataset. Note that we show the target and the prediction corresponding to the input crop which is denoted in *Input* panel by a white dotted rectangle.

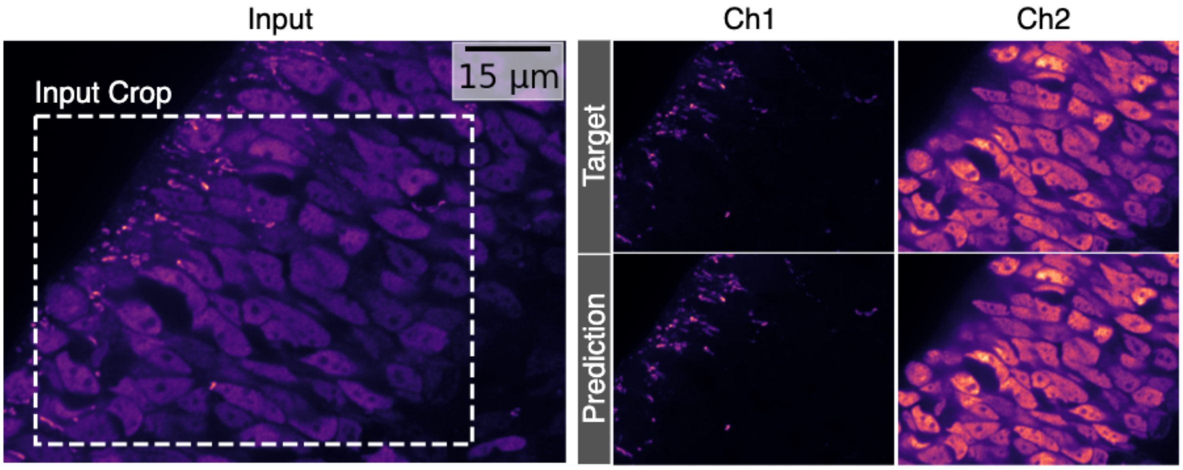

Fig. 31 Qualitative Evaluation for Task XIII from HT-T24 dataset. Note that we show the target and the prediction corresponding to the input crop which is denoted in *Input* panel by a white dotted rectangle.

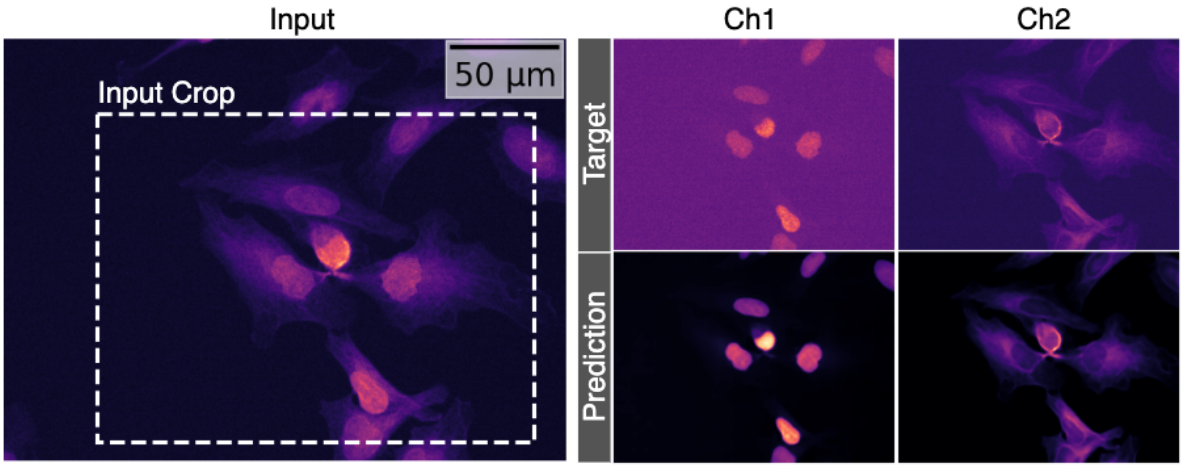

Fig. 32 Qualitative Evaluation for Task XIV from HT-LIF24 dataset. Note that we show the target and the prediction corresponding to the input crop which is denoted in *Input* panel by a white dotted rectangle.

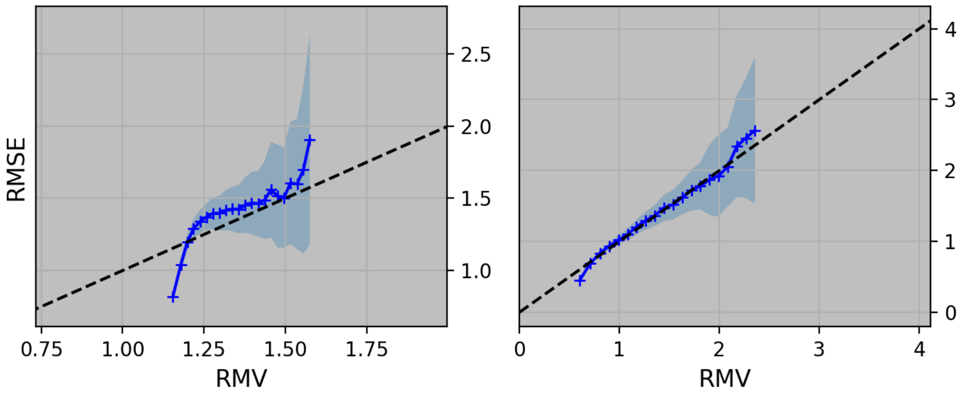

Fig. 33 **Calibration plot for Task XIV from Dataset HT-LIF24**

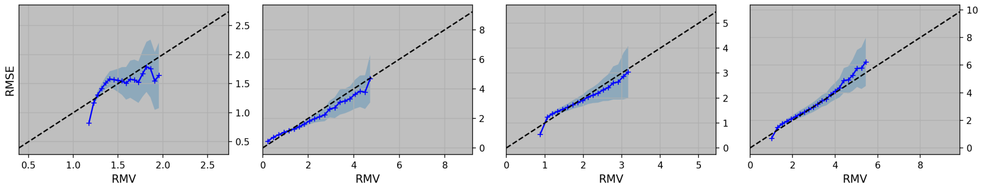

Fig. 34 **Calibration plot for Task XXVIII from Dataset HT-LIF24**

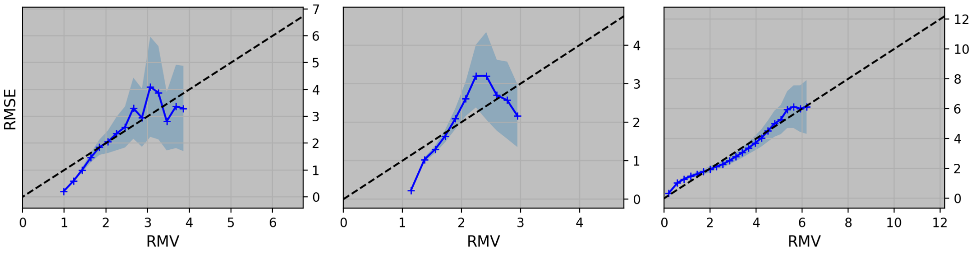

Fig. 35 **Calibration plot for Task XXVI from Dataset HT-LIF24**

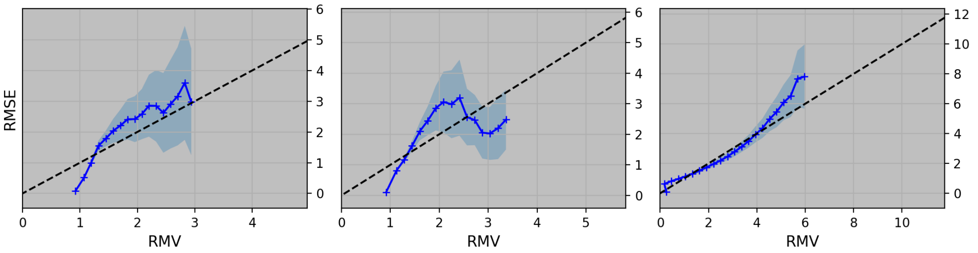

Fig. 36 **Calibration plot for Task XXVII from Dataset HT-LIF24**

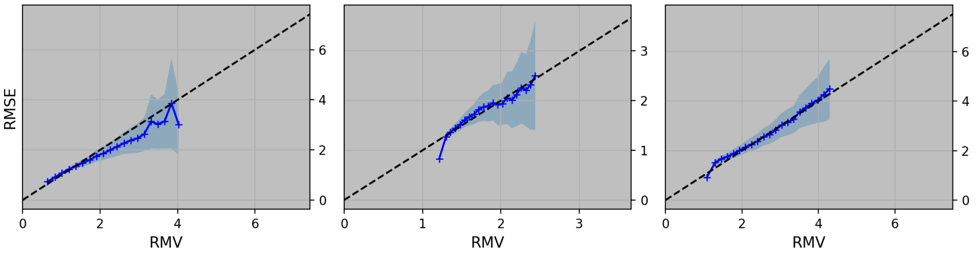

Fig. 37 **Calibration plot for Task XXIII from Dataset HT-LIF24 2ms**

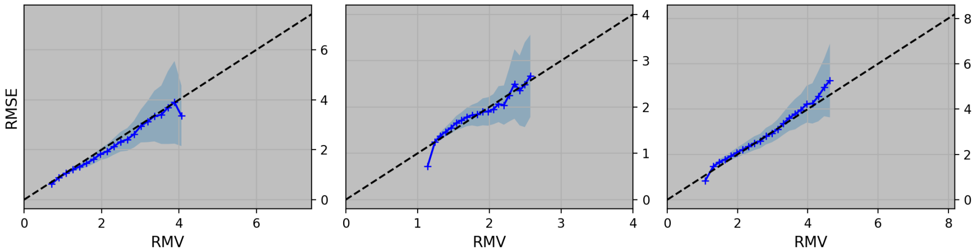

Fig. 38 Calibration plot for Task XXIV from Dataset HT-LIF24

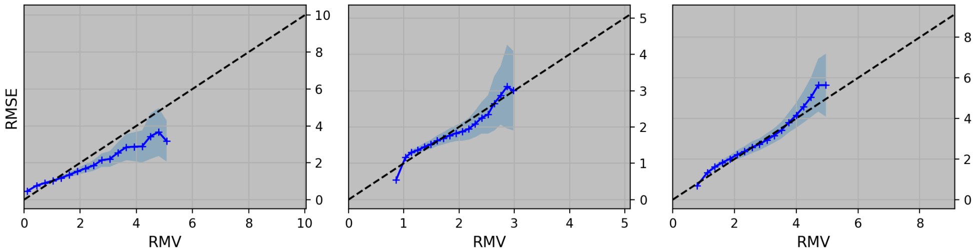

Fig. 39 Calibration plot for Task XXV from Dataset HT-LIF24

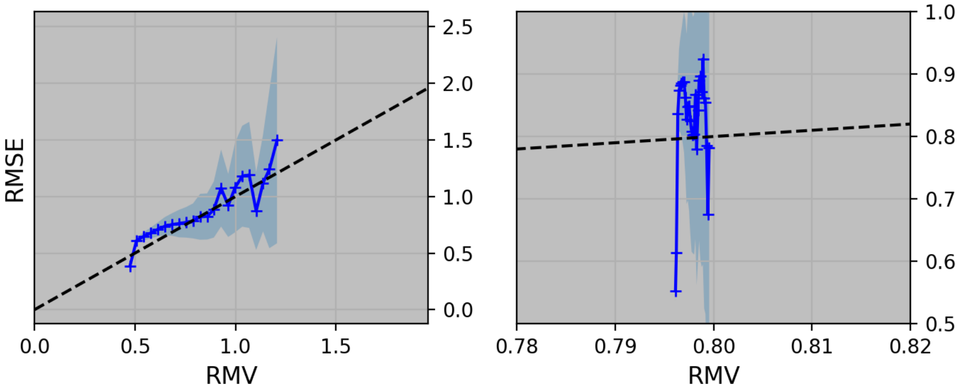

Fig. 40 Calibration plot for Task V from Dataset Pavia-P24, mediumskew, high

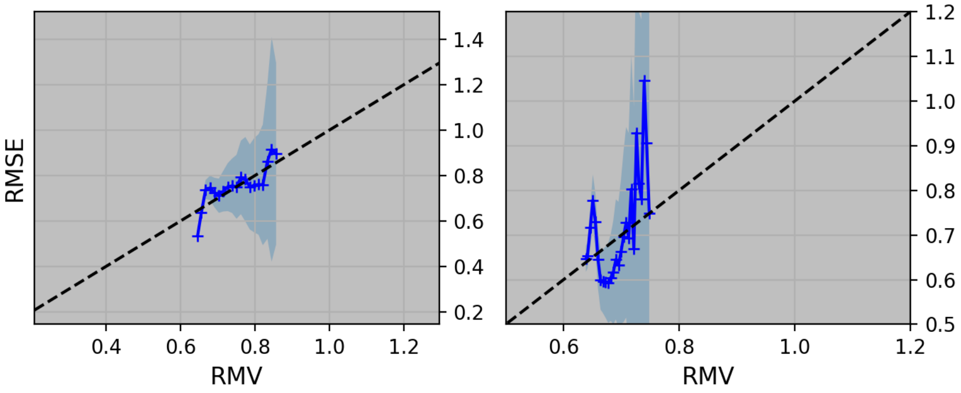

Fig. 41 Calibration plot for Task VII from Dataset Pavia-P24, balanced, medium

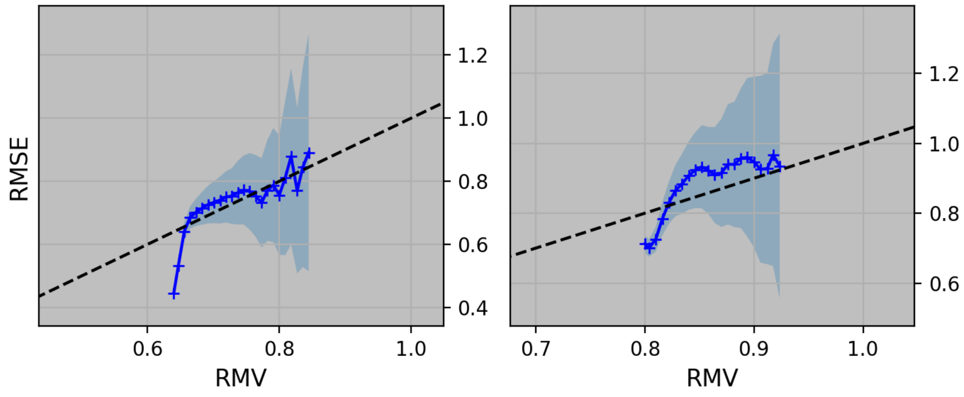

Fig. 42 Calibration plot for Task VIII from Dataset Pavia-P24, mediumskew, medium

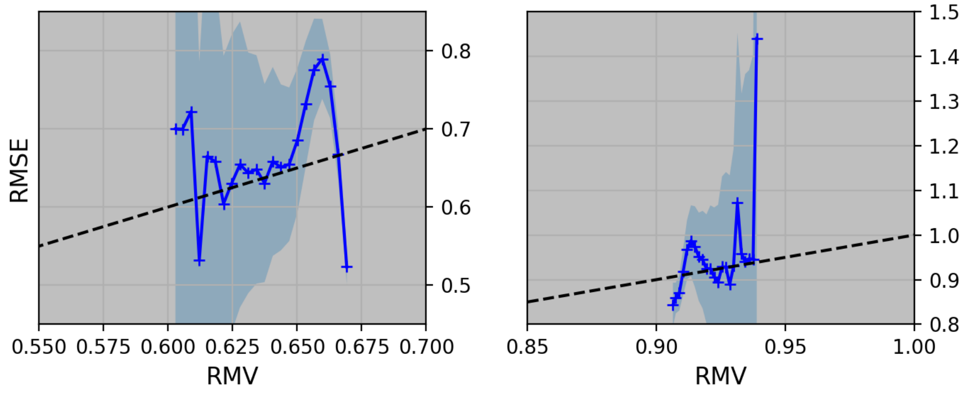

Fig. 43 Calibration plot for Task IX from Dataset Pavia-P24, highskew medium

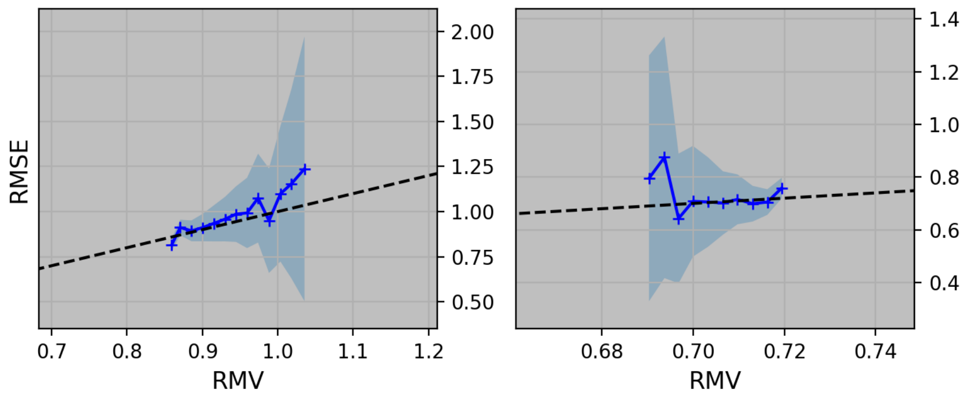

Fig. 44 Calibration plot for Task X from Dataset Pavia-P24, balanced low

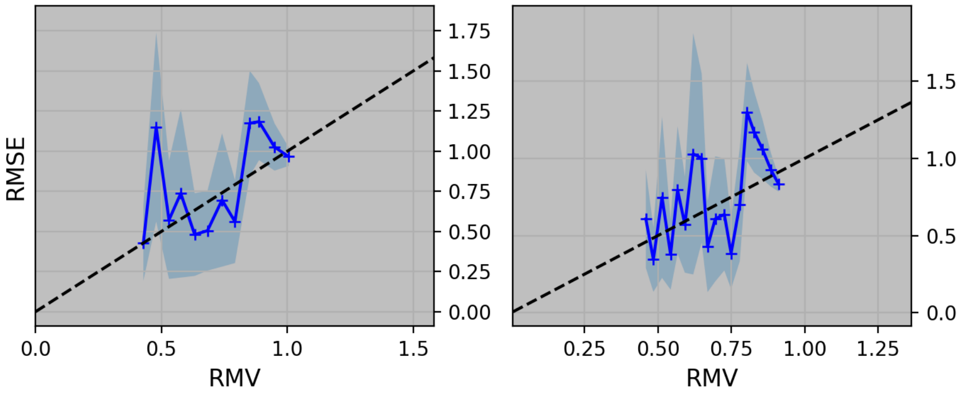

Fig. 45 **Calibration plot for Task XI from Dataset Pavia-P24, mediumskew, low**

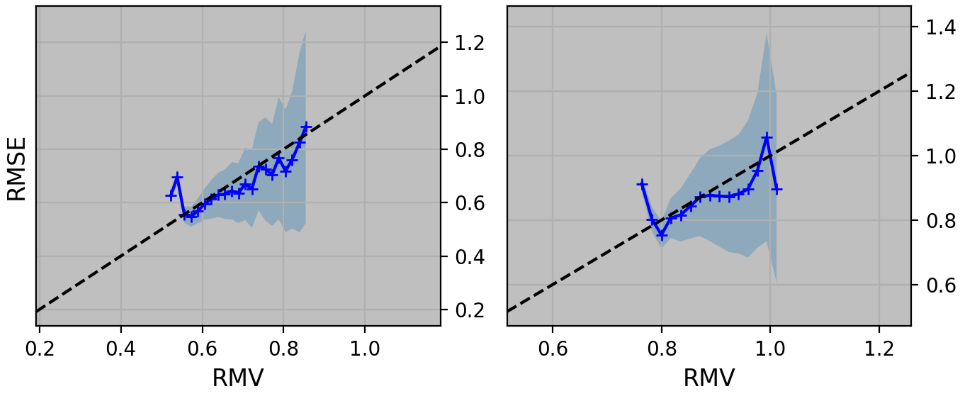

Fig. 46 Calibration plot for Task XII from Dataset Pavia-P24, highskew, low

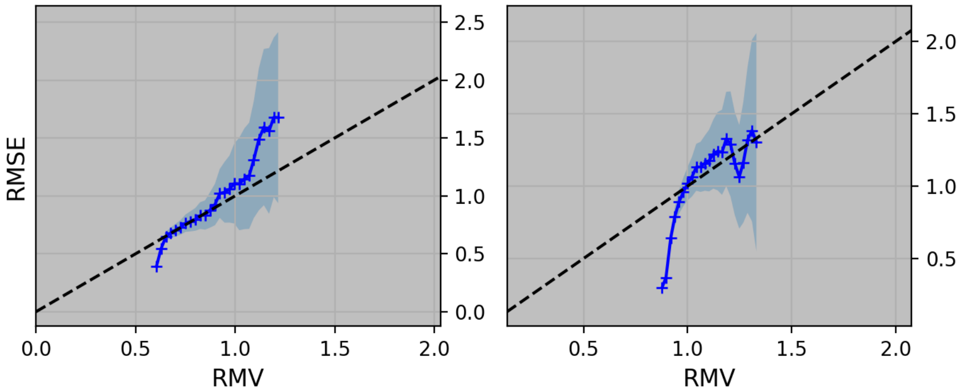

Fig. 47 Calibration plot for Task XX from Dataset Pavia-P24, balanced, high

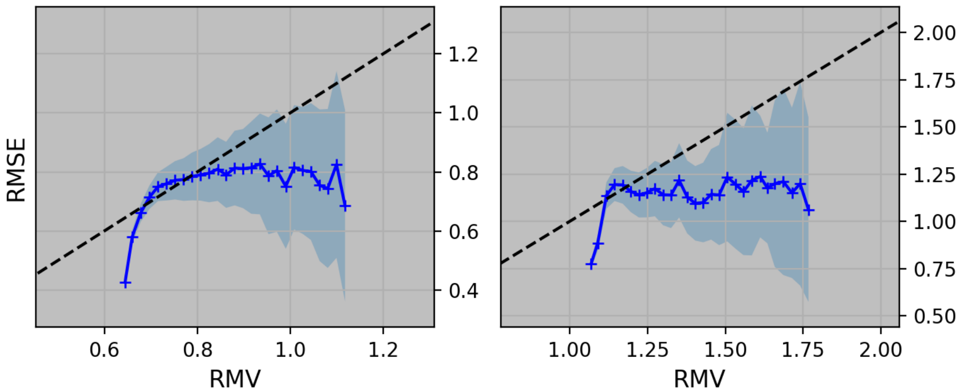

Fig. 48 Calibration plot for Task IV from Dataset Pavia-P24, highskew, high

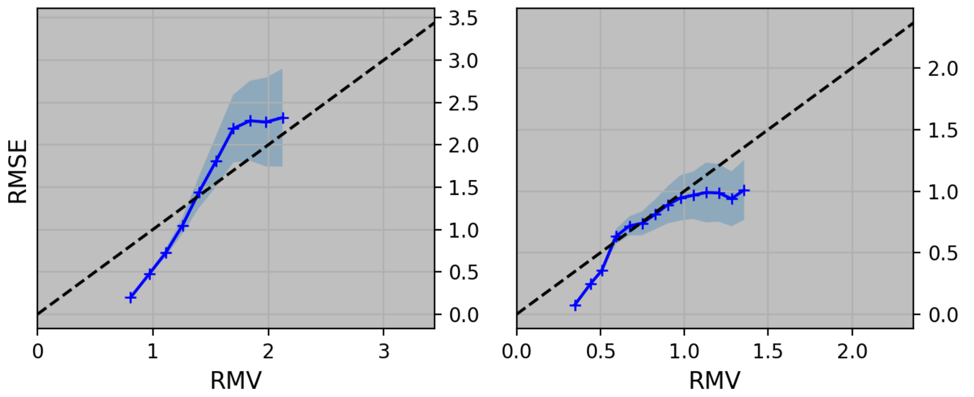

Fig. 49 **Calibration plot for Task I from Dataset HT-H24**

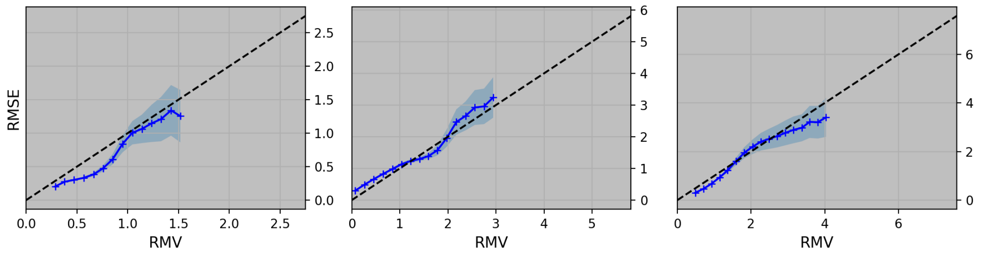

Fig. 50 **Calibration plot for Task XXI from Dataset CBZ-Z18**

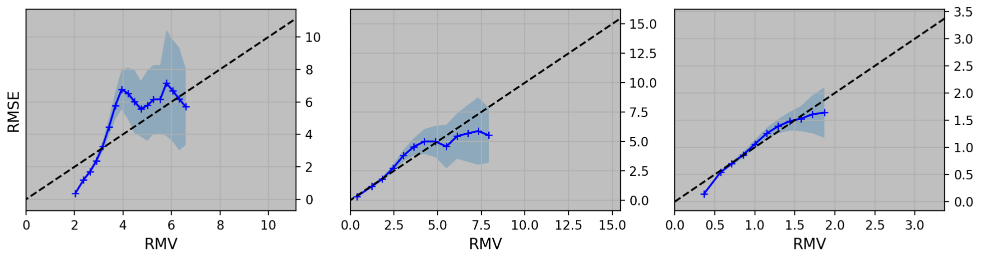

Fig. 51 **Calibration plot for Task XXII from Dataset CBZ-N18**

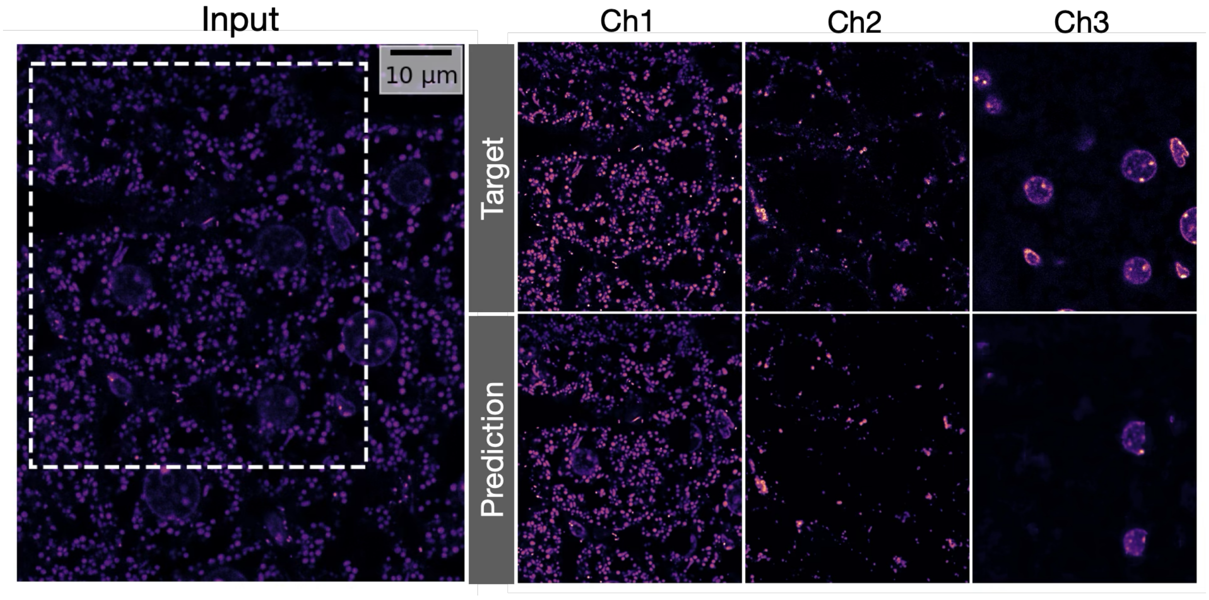

Fig. 52 Qualitative Evaluation for Task XXIII from HHMI-D25$_{8bit}$ dataset. Note that we show the target and the prediction corresponding to the input crop which is denoted in *Input* panel by a white dotted rectangle. Also note that the predictions for channel 3 are of rather poor quality and that you can find a description of how this problem was solved in the Supplementary Section 1.

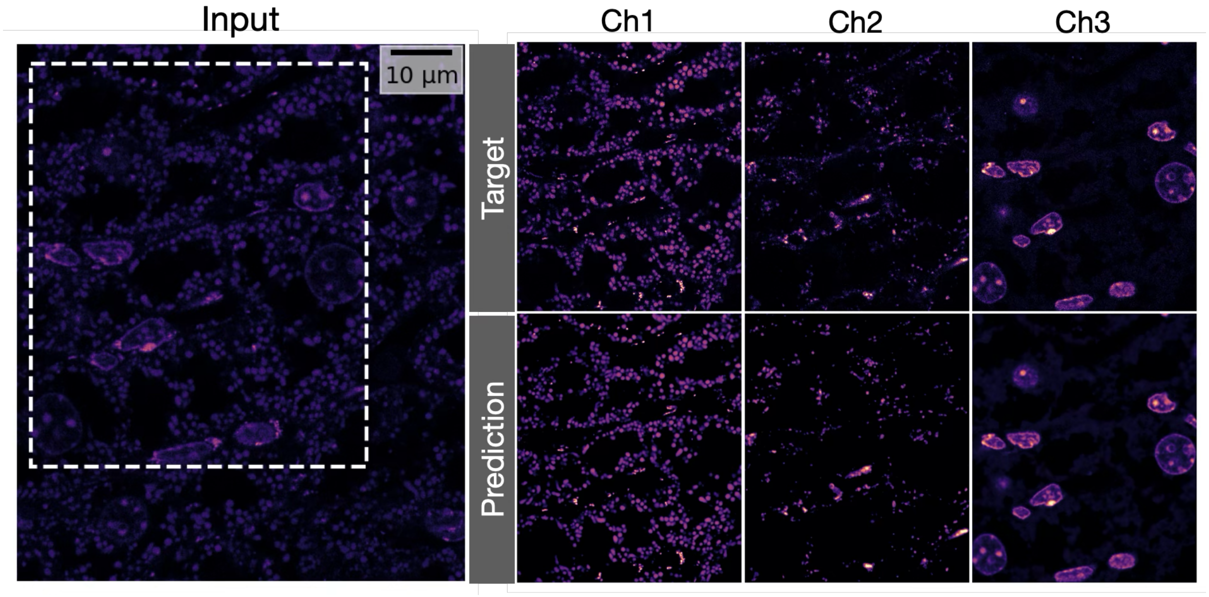

Fig. 53 Qualitative Evaluation for Task XXXIII from HHMI-D25$_{16bit}$ dataset. Note that we show the target and the prediction corresponding to the input crop which is denoted in *Input* panel by a white dotted rectangle.

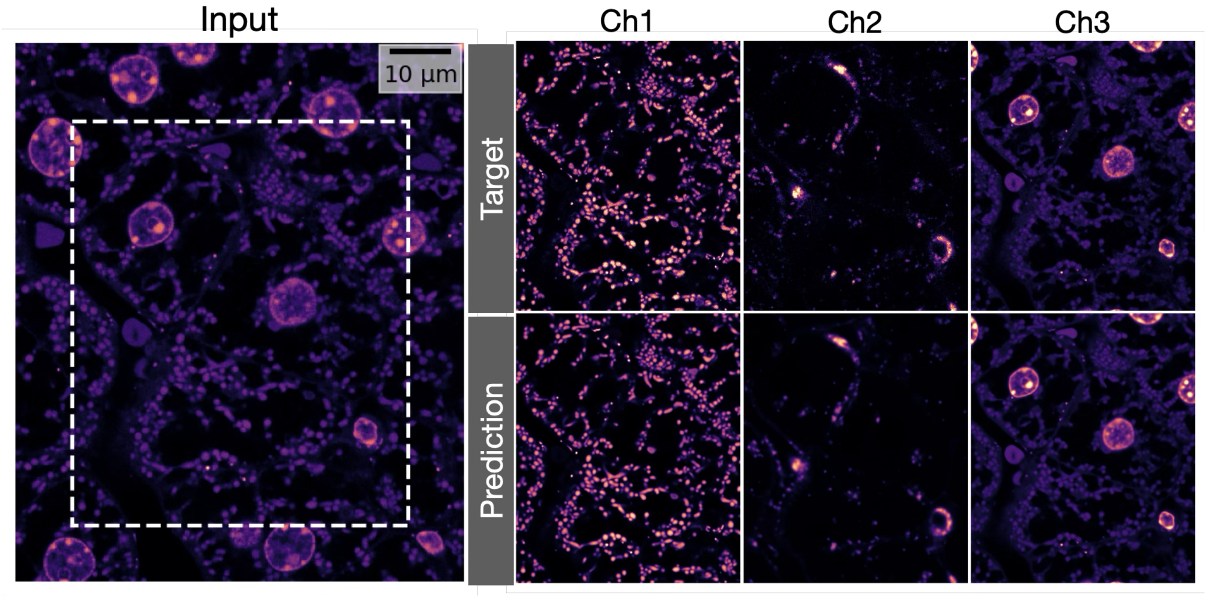

Fig. 54 Qualitative Evaluation for Task XXXVI from HHMI-D25$_{16bit,0.25}$ dataset. Note that we show the target and the prediction corresponding to the input crop which is denoted in *Input* panel by a white dotted rectangle.
