# Supplementary material for: Micro𝕊plit: semantic unmixing of fluorescent microscopy data
Source: Nat Methods. 2026 May 5;23(5):1047–57. doi: 10.1038/s41592-026-03082-1 (PMC13167470; doi:10.1038/s41592-026-03082-1)
Supplement: Supplementary file 2 — Reporting Summary [file 41592_2026_3082_MOESM2_ESM.pdf]

Corresponding author(s): Florian Jug
Last updated by author(s): Dec 19, 2025

Reporting Summary

Nature Portfolio wishes to improve the reproducibility of the work that we publish. This form provides structure for consistency and transparency in reporting. For further information on Nature Portfolio policies, see our Editorial Policies and the Editorial Policy Checklist.

Statistics

For all statistical analyses, confirm that the following items are present in the figure legend, table legend, main text, or Methods section.

n/a	Confirmed	
☐	☒	The exact sample size (*n*) for each experimental group/condition, given as a discrete number and unit of measurement
☐	☒	A statement on whether measurements were taken from distinct samples or whether the same sample was measured repeatedly
☐	☒	The statistical test(s) used AND whether they are one- or two-sided<br>*Only common tests should be described solely by name; describe more complex techniques in the Methods section.*
☒	☐	A description of all covariates tested
☐	☒	A description of any assumptions or corrections, such as tests of normality and adjustment for multiple comparisons
☐	☒	A full description of the statistical parameters including central tendency (e.g. means) or other basic estimates (e.g. regression coefficient) AND variation (e.g. standard deviation) or associated estimates of uncertainty (e.g. confidence intervals)
☒	☐	For null hypothesis testing, the test statistic (e.g. *F*, *t*, *r*) with confidence intervals, effect sizes, degrees of freedom and *P* value noted<br>*Give P values as exact values whenever suitable.*
☒	☐	For Bayesian analysis, information on the choice of priors and Markov chain Monte Carlo settings
☒	☐	For hierarchical and complex designs, identification of the appropriate level for tests and full reporting of outcomes
☒	☐	Estimates of effect sizes (e.g. Cohen's *d*, Pearson's *r*), indicating how they were calculated

Our web collection on statistics for biologists contains articles on many of the points above.

Software and code

Policy information about availability of computer code

Data collection	Most of the data was acquired for this work, a few training tasks are derived from the CARE (NatMeth 2018) work. We have listed all datasets and linked to them here:<br>https://github.com/CAREamics/MicroSplit-reproducibility?tab=readme-ov-file#links-to-all-datasets-used-in-the-manuscript
Data analysis	Results reproducibility notebooks: https://github.com/CAREamics/MicroSplit-reproducibility<br>CAREamics library (fully open): https://careamics.github.io/0.1/

For manuscripts utilizing custom algorithms or software that are central to the research but not yet described in published literature, software must be made available to editors and reviewers. We strongly encourage code deposition in a community repository (e.g. GitHub). See the Nature Portfolio guidelines for submitting code & software for further information.

Data

Policy information about availability of data

All manuscripts must include a data availability statement. This statement should provide the following information, where applicable:

- Accession codes, unique identifiers, or web links for publicly available datasets
- A description of any restrictions on data availability
- For clinical datasets or third party data, please ensure that the statement adheres to our policy

All data used in the manuscript is public and can be found here: https://github.com/CAREamics/MicroSplit-reproducibility?tab=readme-ov-file#links-to-all-datasets-used-in-the-manuscript

Research involving human participants, their data, or biological material

Policy information about studies with human participants or human data. See also policy information about sex, gender (identity/presentation), and sexual orientation and race, ethnicity and racism.

Reporting on sex and gender	No human data was used in this work.
Reporting on race, ethnicity, or other socially relevant groupings	No human data was used in this work.
Population characteristics	No human data was used in this work.
Recruitment	No human data was used in this work.
Ethics oversight	No human data was used in this work.

Note that full information on the approval of the study protocol must also be provided in the manuscript.

Field-specific reporting

Please select the one below that is the best fit for your research. If you are not sure, read the appropriate sections before making your selection.

☒ Life sciences ☐ Behavioural & social sciences ☐ Ecological, evolutionary & environmental sciences

For a reference copy of the document with all sections, see nature.com/documents/nr-reporting-summary-flat.pdf

Life sciences study design

All studies must disclose on these points even when the disclosure is negative.

Sample size	Does not apply. We used diverse datasets to convince readers that our method generally applicable.
Data exclusions	No data was excluded.
Replication	All results have been reproduced multiple times and can be reproduced using our open datasets and open source code.
Randomization	I think this does not apply. We used in our data loaders random sampling of ROIs being feed to the network during network training, but I doubt this is the randomization this form is asking about.
Blinding	Does not apply.

Reporting for specific materials, systems and methods

We require information from authors about some types of materials, experimental systems and methods used in many studies. Here, indicate whether each material, system or method listed is relevant to your study. If you are not sure if a list item applies to your research, read the appropriate section before selecting a response.

Materials & experimental systems

n/a	Involved in the study
☐	☒ Antibodies
☐	☒ Eukaryotic cell lines
☒	☐ Palaeontology and archaeology
☐	☒ Animals and other organisms
☒	☐ Clinical data
☒	☐ Dual use research of concern
☒	☐ Plants

Methods

n/a	Involved in the study
☒	☐ ChIP-seq
☒	☐ Flow cytometry
☒	☐ MRI-based neuroimaging

Antibodies

Antibodies used

1. Puncta (HT-H23): (does not name it, but says "a secondary antibody conjugated to a 555 dye")
2. Pigino lab: anti alpha-tubulin (ABCD antibodies AA_345), anti-beta tubulin (AA_344) Mouse IgG2a raised antibody, Guinea Pig IgG raised antibody, Goat Anti-Mouse IgG Secondary Antibody, donkey anti-Rabbit IgG(H+L) Highly Cross-Adsorbed Secondary Antibody.
3. HT-T24: S*OX2 (goat polyclonal, AF2018, 1:200, R&D Systems) and GRASP65 (rabbit polyclonal, PA3-910, 1:200, Invitrogen);* donkey anti-Goat IgG(H+L) Highly Cross-Adsorbed Secondary Antibody, Alexa Fluor Plus 555 (A32816, 1:500, Invitrogen) and donkey anti-Rabbit IgG(H+L) Highly Cross-Adsorbed Secondary Antibody, Alexa Fluor Plus 647 (A32795, 1:500, Invitrogen).
4. Feliciano lab: Mouse anti-PMP70 (MilliporeSigma, SAB4200181, 1:75), rabbit anti-LAMP1 (Abcam, AB208943, 1:50), Alexa Fluor 647 goat anti-mouse antibody (Thermo Fisher, A21235, 1:500), Alexa Fluor 750 goat anti-rabbit antibody (Thermo Fisher, A21039, 1:500)
5. HT-LIF24: anti-α-tubulin mouse IgG monoclonal primary antibody (T5168, Sigma-Aldrich; 1:100); anti-laminin B1 rabbit IgG polyclonal primary antibody (ab16048, Abcam; 1:200); anticentromere protein human IgG polyclonal primary antibody (15-234, Antibodies Incorporated; 1:400); Alexa Fluor 488 donkey anti-mouse IgG secondary antibody (A-21202, Thermo Fisher Scientific; 1:400); Cy3 donkey anti-rabbit IgG secondary antibody (711-165-152, Jackson Immunoresearch; 1:400); Cy5 goat anti-human IgG secondary antibody (109-175-088, Jackson Immunoresearch; 1:50).
6. HT_H23 dataset. Primary antibody = Rat α CTIP2 1:500 (1:500, Abcam, ab18465). Secondary antibody = Donkey α Rat Alexa Fluor™ Plus 555 (1:1000, Invitrogen, A48270)
7. HT_H24 dataset: Primary antibodies = Rat α SOX2 (1:200, Invitrogen, 14-9811-82), Chicken α MAP2 (1:5000, Invitrogen, PA1-10005). Secondary antibodies = Donkey α Chicken Alexa Fluor 488 (1:1000, Jackson ImmunoResearch, 703-545-155), Donkey α Rat Alexa Fluor™ Plus 555 (1:1000, Invitrogen, A48270).

Validation

No validation. For this work, this was not relevant.

Eukaryotic cell lines

Policy information about cell lines and Sex and Gender in Research

Cell line source(s)

HT_P23A and HT_P23B datasets from Pigino lab: MDCK-II ccll line

HT-LIF24 dataset: HeLa cell line (tested negative for Mycoplasma), no authentication needed.

HT_H23 dataset: WA-09 human pluripotent stem cells (hPSCs)

HT_H24 dataset: WTC-11 (UCSFi001-A) induced pluripotent stem cells (iPSCs)

Pavia-P23 dataset: HaCaT cell line

Chicago-Sch23 dataset: Human BJ fibroblast cell line

Authentication

Describe the authentication procedures for each cell line used OR declare that none of the cell lines used were authenticated.

Mycoplasma contamination

HT_P23A, HT_P23B from Pigino lab: MDCK-II: mycoplasma free certification, regular testing.

HT-LIF24: HeLa cell line, tested negative for Mycoplasma.

Pavia-P23: tested for mycoplasma and bacterial contamination twice a year.

Commonly misidentified lines
(See ICLAC register)

Name any commonly misidentified cell lines used in the study and provide a rationale for their use.

Animals and other research organisms

Policy information about studies involving animals; ARRIVE guidelines recommended for reporting animal research, and Sex and Gender in Research

Laboratory animals

HT-T24 (Ferret (Mustela furo))

HHMI-D25, Feliciano Lab: Heterozygous PhamExcised females carrying the Mito_Dendra2 transgene obtained by crossing PhamExcised males (strain #018397 derived homozygous males) from Jackson Laboratories and wild type C57BL/6J females

Wild animals

Provide details on animals observed in or captured in the field; report species and age where possible. Describe how animals were caught and transported and what happened to captive animals after the study (if killed, explain why and describe method; if released, say where and when) OR state that the study did not involve wild animals.

Reporting on sex

HT-T24 (A pregnant female was sacrificed to take the embryos whose sex was unknown, because they were at an early stage to determine it.)

HHMI-D25: In this study, we developed Microsplit, a computational multiplexing technique based on deep learning that allows multiple cellular structures to be imaged in a single fluorescent channel and then unmix them by computational means. Although gender was not the focus of this study and not a critical factor, HHMI-D25 data sets were acquired from female mice due to their availability at the time of experiment.

Field-collected samples

For laboratory work with field-collected samples, describe all relevant parameters such as housing, maintenance, temperature, photoperiod and end-of-experiment protocol OR state that the study did not involve samples collected from the field.

Ethics oversight

HT-T24: Animals used for this study were kept in standardized hygienic conditions at the Biomedical Services Facility (BMS) of the MPI-CBG with free access to food and water. All experimental procedures were conducted in agreement with the German Animal Welfare Legislation after approval by the Landesdirektion Sachsen (license for ferret TVV2/2015

HHMI-D25, Feliciano Lab: All animal care and procedures were conducted according to NIH guidelines and were approved by the Institutional Animal Care and Use Committee (Protocol #22-0229.04) at Janelia Research Campus, Howard Hughes Medical Institute.

Note that full information on the approval of the study protocol must also be provided in the manuscript.

Plants

Seed stocks

Report on the source of all seed stocks or other plant material used. If applicable, state the seed stock centre and catalogue number. If plant specimens were collected from the field, describe the collection location, date and sampling procedures.

Novel plant genotypes

Describe the methods by which all novel plant genotypes were produced. This includes those generated by transgenic approaches, gene editing, chemical/radiation-based mutagenesis and hybridization. For transgenic lines, describe the transformation method, the number of independent lines analyzed and the generation upon which experiments were performed. For gene-edited lines, describe the editor used, the endogenous sequence targeted for editing, the targeting guide RNA sequence (if applicable) and how the editor was applied.

Authentication

Describe any authentication procedures for each seed stock used or novel genotype generated. Describe any experiments used to assess the effect of a mutation and, where applicable, how potential secondary effects (e.g. second site T-DNA insertions, mosiacism, off-target gene editing) were examined.
